# Trans-oceanic genomic divergence of Atlantic cod ecotypes is associated with large inversions

**DOI:** 10.1038/hdy.2017.54

**Published:** 2017-09-20

**Authors:** P R Berg, B Star, C Pampoulie, I R Bradbury, P Bentzen, J A Hutchings, S Jentoft, K S Jakobsen

**Affiliations:** 1 Centre for Ecological and Evolutionary Synthesis, Department of Biosciences, University of Oslo, Oslo, Norway; 2 Centre for Molecular Medicine Norway, Faculty of Medicine, University of Oslo, Oslo, Norway; 3 Marine and Freshwater Research Institute, Reykjavik, Iceland; 4 Department of Fisheries and Oceans, St John’s, Newfoundland, Canada; 5 Ocean Sciences Centre, Memorial University of Newfoundland, St John’s, Newfoundland, Canada; 6 Department of Biology, Dalhousie University, Halifax, Nova Scotia, Canada; 7 Institute of Marine Research, Flødevigen Marine Research Station, His, Norway; 8 Centre for Coastal Research, University of Agder, Kristiansand, Norway

## Abstract

Chromosomal rearrangements such as inversions can play a crucial role in maintaining polymorphism underlying complex traits and contribute to the process of speciation. In Atlantic cod (*Gadus morhua*), inversions of several megabases have been identified that dominate genomic differentiation between migratory and nonmigratory ecotypes in the Northeast Atlantic. Here, we show that the same genomic regions display elevated divergence and contribute to ecotype divergence in the Northwest Atlantic as well. The occurrence of these inversions on both sides of the Atlantic Ocean reveals a common evolutionary origin, predating the >100 000-year-old trans-Atlantic separation of Atlantic cod. The long-term persistence of these inversions indicates that they are maintained by selection, possibly facilitated by coevolution of genes underlying complex traits. Our data suggest that migratory behaviour is derived from more stationary, ancestral ecotypes. Overall, we identify several large genomic regions—each containing hundreds of genes—likely involved in the maintenance of genomic divergence in Atlantic cod on both sides of the Atlantic Ocean.

## Introduction

Genomic islands of divergence (Wu, 2001; Nosil *et al.*, 2009) are known to emerge through divergence hitchhiking (Via, 2012) but can also evolve through other processes that reduce recombination in genomic regions, such as inversions (Kirkpatrick and Barton, 2006). Chromosomal rearrangements in the form of inversions have been hypothesized to play a key role in maintaining polymorphism in complex traits (Conrad and Hurles, 2007). Within inversions, the rate of crossing over is reduced by several orders of magnitude, allowing genomic islands of divergence within inversions to be larger than in collinear regions. If an inversion captures several locally adapted alleles, it can be integral to the genomic process of local adaptation because it suppresses meiotic recombination in heterozygous individuals (Kirkpatrick and Barton, 2006).

Inversion polymorphisms have been linked to adaptation with gene flow in several species including *Drosophila* (Noor *et al.*, 2001), *Helianthus* sunflowers (Rieseberg, 2001), *Anopheles* mosquitoes (Ayala and Coluzzi, 2005) and *Agrodiaetus* butterflies (Kandul *et al.*, 2007). Recently, evidence of genomic islands of divergence caused by chromosomal inversions has been reported in several studies focussing on Atlantic cod (*Gadus morhua* L.) (Berg *et al.*, 2016; Sodeland *et al.*, 2016; Kirubakaran *et al.*, 2016; Barth *et al.*, 2017). Inversions that differentiate migratory from nonmigratory ecotypes (Berg *et al.*, 2016; Kirubakaran *et al.*, 2016) have been documented for cod in the Northeast Atlantic, either in only a small fraction of the genome (Kirubakaran *et al.*, 2016) or a few distinct populations (Berg *et al.*, 2016; Sodeland *et al.*, 2016). Existence of these inversions in the Northwest Atlantic or around Iceland has not yet been investigated, although genomic islands of divergence have previously been documented for several populations (Bradbury *et al.*, 2010, 2013; Hemmer-Hansen *et al.*, 2013; Berg *et al.*, 2015, 2016). The fact that such ‘islands’ have previously been identified on both sides of the Atlantic (Bradbury *et al.*, 2010, 2013) suggests that inversions might also play a role in explaining genomic islands of divergence in Northwest Atlantic and Icelandic cod. Interestingly, the allele frequencies of these ‘islands’ display parallel latitudinal clines in populations that are otherwise genetically distinct, on both sides of the Atlantic (Bradbury *et al.*, 2010), that is indicative of parallel evolution. Furthermore, a subset of the single-nucleotide polymorphisms (SNPs) investigated by Bradbury *et al.* (2010) has also been associated with temperature in several other studies (Nielsen *et al.*, 2009; Hemmer-Hansen *et al.*, 2013; Therkildsen *et al.*, 2013).

Here, we investigate Atlantic cod populations from both sides of the Atlantic Ocean (Table 1) that have previously been partitioned into (1) a northern (Can-N) and a southern (Can-S) group (Bradbury *et al.*, 2010, 2013) in the Northwest Atlantic; (2) Frontal and Coastal ecotypes in Iceland (see, for example, Pampoulie *et al.*, 2008, 2015); and (3) migratory North East Arctic cod (NEAC) and nonmigratory Norwegian coastal cod (NCC) in the Northeast Atlantic. First, we identify outlier SNPs and genomic regions putatively under selection for each population pair and between the spatially different subset of populations within each of the three broadly delineated groups (comparing Can-N with Can-S, Frontal with Coastal and NEAC with NCC), and then make a trans-Atlantic comparison of the observed genomic patterns. Second, we explore linkage disequilibrium (LD) patterns, combined with SNP information, to look for chromosomal rearrangements and to investigate whether previously identified inversions (Berg *et al.*, 2016; Sodeland *et al.*, 2016; Kirubakaran *et al.*, 2016) occur throughout the distribution range of Atlantic cod. Finally, we investigate the frequencies of the inversions among populations, both within and across the continents, to unravel their distributions and likely origins, and discuss possible mechanisms driving the observed patterns in the light of ecotype divergence and adaptation. The results provide insight into the process of genomic divergence in marine fishes in general.

## Materials and methods

### Samples, DNA extraction and genotyping

**Figure 1:**
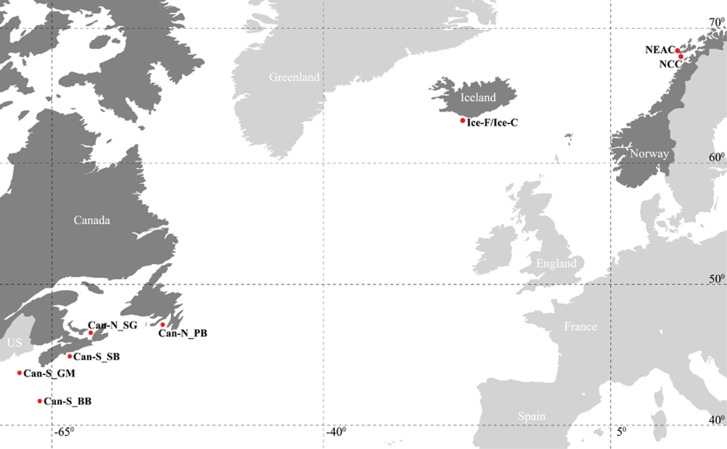
Map showing the sampling locations of the Atlantic cod populations used in the present study. With one exception, red dots indicate the position where the samples were collected; the Icelandic samples were collected at several locations in the waters around Iceland and later categorized as Frontal or Coastal, based on Data Storage Tag profiling. See Figure 1 in Thorsteinsson *et al.* (2012) for a detailed view of the Icelandic sampling localities and see Table 1 for a detailed description of the populations in the present study. Can-N_PB, Placentia Bay; Can-N_SG, Southern Gulf of St Lawrence; Can-S_SB, Sambro; Can-S_GM, Gulf of Maine; Can-S_BB, Browns Bank; Ice_F, Iceland Frontal; Ice_C, Iceland Coastal; NEAC, Northeast Arctic cod; NCC, Norwegian coastal cod. The map was modified from http://www.graphic-flash-sources.com/world-vector-map/ using Adobe Illustrator CC.

We sampled 316 Atlantic cod (Figure 1 and Table 1), consisting of 144 individuals from 5 locations from the Northwest Atlantic, 39 Frontal and 39 Coastal ecotype individuals from Iceland (classified by data storage tag (DST) profiles, see Pálsson and Thorsteinsson, 2003; Thorsteinsson *et al.*, 2012), and 50 NEAC and 44 NCC individuals from the Northeast Atlantic.

DNA was extracted from muscle tissue using the E.Z.N.A Tissue DNA kit (Omega Bio-Tek, Norcross, GA, USA) and normalized to 100 ng μl^−1^. All samples were individually genotyped using a 12K Illumina SNP chip for which 8165 SNPs were polymorphic in this data set, had a call rate of >95% and showed Mendelian inheritance in a separate set of individuals with a pedigree. Out of these SNPs, 602 were close to selected candidate genes, 1470 were nonsynonymous SNPs and the remaining 5857 SNPs were randomly distributed throughout the 23 different linkage groups (LGs). Genotype clustering was performed in Genome Studio 2011.1 (Illumina Inc., San Diego, CA, USA). The nomenclature of the LGs follows Hubert *et al.* (2010) and the order of the SNPs are as in Berg *et al.* (2016). All 8165 SNPs used were mapped to the published Atlantic cod genome (ATLCOD1C) (Star *et al.*, 2011) in the same way as in Berg *et al.* (2016) and details are available in dbSNP (www.ncbi.nlm.nih.gov/snp).

### Outlier detection and population genetics

Allele frequencies and observed and expected heterozygosity (*H*_o_ and *H*_e_) within each population were calculated in ARLEQUIN 3.5.1.3 (Excoffier and Lischer, 2010). Departure from Hardy–Weinberg equilibrium was tested locus by locus in each population in ARLEQUIN with 100 000 iterations and a Markov Chain of 1 000 000. Correction for multiple testing was performed in *R* (R Core Team, 2012), using the QVALUE package (Storey, 2002) with a *q*-value of 0.05 as a threshold.

Outlier detection in the respective data sets was performed using 10 independent runs of BAYESCAN v2.1 (Foll and Gaggiotti, 2008), using stringent criteria, assuming selection to be 10% and false discovery rate set to 0.01. We report both the median log_10_(posterior odds) and the median *q*-value. As outlier tests may have a high rate of false positives because of the effects of population demography and bottlenecks (Narum and Hess, 2011; de Villemereuil *et al.*, 2014), and because of the clear trans-Atlantic divergence in the data, we performed outlier analyses pairwise between all population pairs or identified groups to reduce the methodological weakness caused by population structuring (Vitalis *et al.*, 2001).

Based on the outlier analyses, SNPs were categorized as outliers or as neutral. To avoid bias in the *F*_ST_ and STRUCTURE (Pritchard *et al.*, 2000) analyses, tag SNPs based on LD values between SNPs (*r*^2^>0.5) were selected using PLINK v1.07 (Purcell *et al.*, 2006). The outlier and neutral data sets (518 and 7369 SNPs) are represented by 325 and 7075 unlinked tag-SNPs. Locus-specific *F*_ST_ values and weighted average *F*_ST_ values between all populations were calculated in ARLEQUIN, using 10 000 permutations. We calculated nucleotide diversity (*π*) within all identified groups, and nucleotide divergence between these groups (*D*_*XY*_), using a sliding windows approach with a 50-SNP window and 10 SNPs per iteration in DnaSP 5.10 (Librado and Rozas, 2009). These analyses were also performed locally within each of the identified chromosomal rearrangements.

Discriminant analysis of principal components (DAPC), using all 8165 SNPs were performed, using the *R* package ADEGENET (Jombart and Ahmed, 2011). The correlated allele frequency and admixture model in STRUCTURE was used to identify major genetic clusters in the data set, performing 10 independent runs for each value of *K* (burn-in of 10 000 Markov chain Monte Carlo iterations followed by 100 000 iterations) on the different data sets. Delta *K* and the best *K*-value for each data set in STRUCTURE was identified with CLUMPAK (Kopelman *et al.*, 2015). NETVIEW P (Steinig *et al.*, 2016) was used to visualize the neutral population divergence in the data based on an isolation by state (1−IBS) matrix constructed in PLINK at *k*=50, using 7075 unlinked neutral SNPs. The network construction is independent of prior population information and based solely on the genetic distance between individuals.

### LD and rearrangement patterns

The presence of intrachromosomal LD, quantified with the *r*^2^ estimate using PLINK, was evaluated in all populations separately and within the identified groups (Table 1). The *R* package inveRsion (Cáceres *et al.*, 2012) was used to detect and locate potentially inverted genomic regions and to identify the inversion status of each individual, using block size=3, min. allele=0.1 and thbic=0. This method utilizes the LD differences across inversion breakpoints to detect potentially inverted regions. To complement this LD-based approach, the *R* package invClust (Cáceres and González, 2015) was also used to identify potentially inverted regions by haplotype tagging and dimensionality reduction analysis based on predefined regions of interest. These regions were defined based on the LD analyses performed in PLINK. DAPCs were performed within the identified inversions to visualize the distinct three-cluster pattern, reflecting the different inversion genotypes. Simulation analyses have demonstrated that such analyses can be used efficiently to detect and genotype inversion polymorphisms of unphased SNP data (Ma and Amos, 2012).

## Results

We investigated a total of 8165 SNPs, distributed throughout 23 LGs with an average distance of 94 000 bp between SNPs, based on a genome size of 830 Mb (Star *et al.*, 2011), in 316 individuals of cod from both sides of the Atlantic Ocean (Figure 1 and Table 1). A total of 5202 SNPs were located within 5000 bp of 4245 Ensembl annotated genes. Only seven SNP loci were significantly out of Hardy–Weinberg Equilibrium, after false discovery rate correction (*q*<0.05), in any of the populations (Supplementary Table S1), indicating no Wahlund effect. The number of polymorphic loci and the observed and expected heterozygosity was generally lower in Northwest Atlantic populations than in Northeast Atlantic populations (Table 1).

### Population divergence

Neutral weighted *F*_ST_ between the Northwest and Northeast Atlantic was 0.081 and all pairwise *F*_ST_ values were significantly different from zero except for the Can-S_BB comparisons with Can-S_SB and Can-S_GM and the Ice_F/Ice_C comparison (Supplementary Table S2; see Table 1 for sample codes). The *F*_ST_ values based on the outlier SNPs (see below) were generally orders of magnitude larger than those based on the neutral SNPs in any pairwise comparison and only the Can-S_BB comparisons with Can-S_SB and Can-S_GM were not significantly different from zero. Elevated *F*_ST_ values predominantly occurred within distinct regions in LGs 2, 7 and 12 (but also to some extent in LG1) in the Northwest Atlantic populations, primarily in LGs 1, 2 and 7 in the Northeast Atlantic populations, and in a distinct region in LG23 and in a few SNPs in LG11 between the two continents (Supplementary Figure S1). This pattern corresponds well with the distinctly different heterozygosity and nucleotide divergence (*D*_*XY*_) patterns observed (Supplementary Figures S2 and S3).

**Figure 2:**
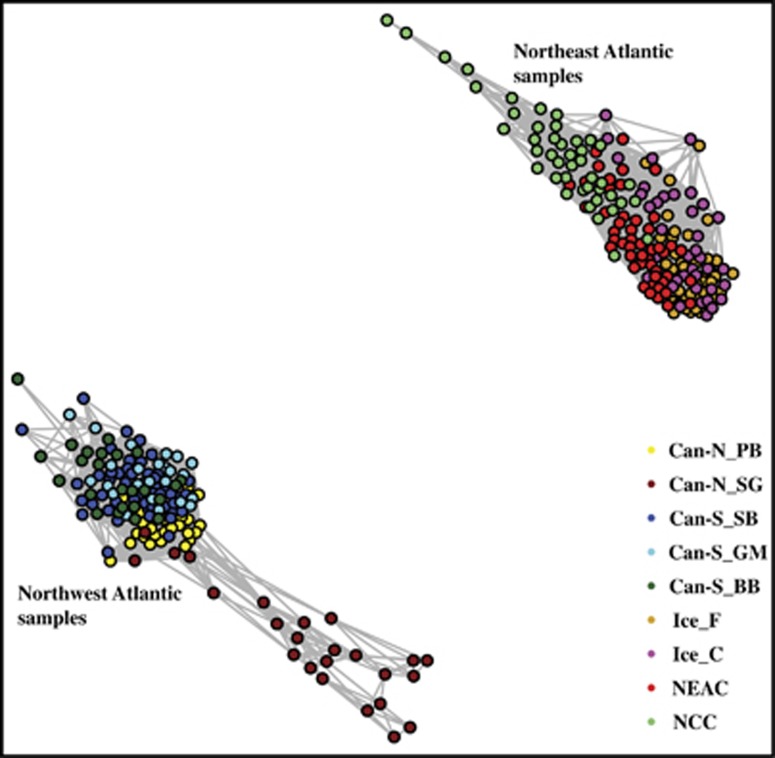
Neutral population divergence between Atlantic cod populations. Population clustering based on 7075 neutral SNPs in 316 individuals of Atlantic cod, using an isolation-by-state (IBS) matrix constructed in PLINK, visualized using the NETVIEW P pipeline at *k*=50, capturing large-scale genetic differentiation across the Atlantic as well as fine-scale structuring within the Northwest and Northeast Atlantic populations. Edge width is proportional to the genetic distance between individuals. Can-N_PB, Placentia Bay; Can-N_SG, Southern Gulf of St Lawrence; Can-S_SB, Sambro; Can-S_GM, Gulf of Maine; Can-S_BB, Browns Bank; Ice_F, Iceland Frontal; Ice_C, Iceland Coastal; NEAC, Northeast Arctic cod; NCC, Norwegian coastal cod.

**Figure 3:**
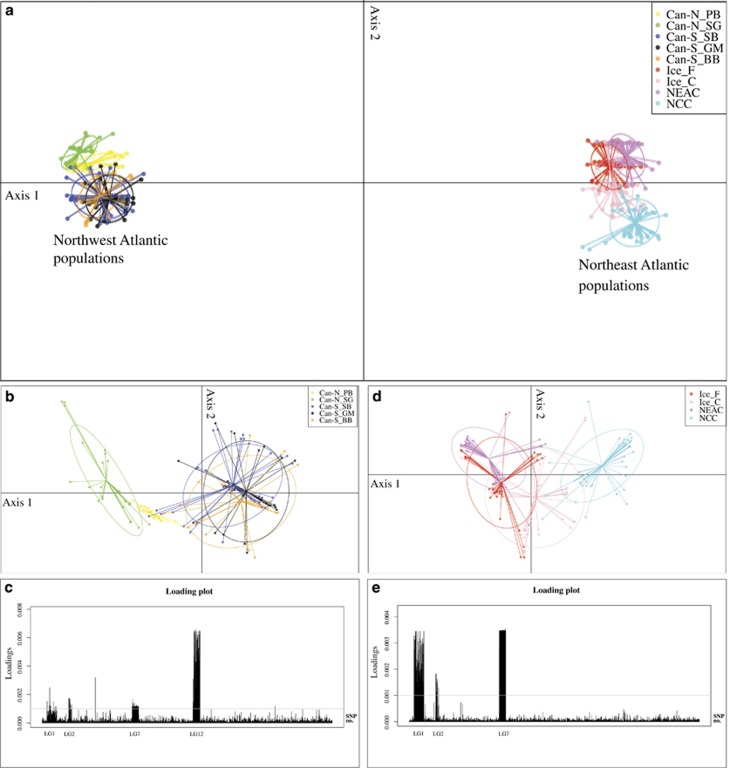
Spatial relationship between and within Northwest and Northeast Atlantic cod based on discriminant analysis of principal components (DAPC). Based on all 8165 SNPs, a distinct trans-Atlantic separation and a clear separation within Northeast and Northwest Atlantic is observed (**a**). The stratification within the Northwest Atlantic (**b**) and the Northeast Atlantic (**d**) is even more evident when these groups are analysed separately. The loading plots based on the DAPC analyses show the contribution of each SNP to the differentiation within the Northwest Atlantic (**c**) and Northeast Atlantic (**e**) populations. The analyses are based on n.pca=3 and n.da=2, calculated in ADEGENET that assumes a predefined population designation of the individuals. Can-N_PB, Placentia Bay; Can-N_SG, Southern Gulf of St Lawrence; Can-S_SB, Sambro; Can-S_GM, Gulf of Maine; Can-S_BB, Browns Bank; Ice_F, Iceland Frontal; Ice_C, Iceland Coastal; NEAC, Northeast Arctic cod; NCC, Norwegian coastal cod.

Bayesian cluster analyses as implemented in STRUCTURE supported a distinct separation (Δ*K*=2) between Northwest and Northeast Atlantic populations, using both the neutral and the outlier data sets (Supplementary Figure S4). In addition, the STRUCTURE analysis based on the neutral data set (*K*=3) revealed that the NCC population is distinctly different from the other Northeast Atlantic populations. Further neutral population structuring within both the Northwest and Northeast Atlantic was evident from the network analyses (Figure 2). The DAPC, using all SNPs, confirmed the distinct separation between Northwest and Northeast Atlantic populations (Figure 3a), and also revealed a further stratification within these regions (Figures 3b and d). Within the Northwest Atlantic, the Can-S_SB clustered together with the Can-S populations that clustered separately from the Can-N populations (Figure 3b). Within the Northeast Atlantic, the Frontal and Coastal ecotypes from Iceland clustered closer to the migratory NEAC population, whereas the nonmigratory NCC population was distinctly different from these. When grouping the populations into Can-N/migratory and Can-S/non-migratory entities (Table 1), we observed clear genomic differences that could be attributed to the putative inversions within LGs 1, 2, 7 and 12 (Supplementary Figures S5a and b). Moreover, the separation pattern within the different LGs primary reflected the frequency differences between these regions (Figures 3c and e, Supplementary Figures S5c–f). The remaining LGs showed little differentiation between these two groups (Supplementary Figure S5g).

### Outlier detection and identification of genomic regions under selection

**Figure 4:**
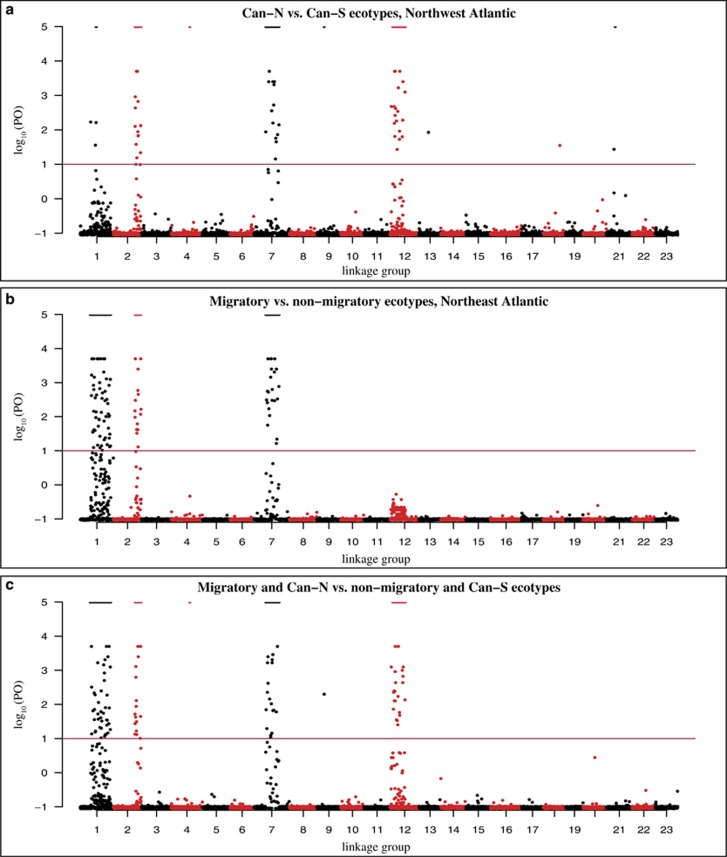
Manhattan plots visualizing the pairwise outlier patterns between ecotypes of Atlantic cod. The observed outlier pattern between Can-N and Can-S (**a**) indicates that the majority of outliers are clustered within the inversions in LGs 2, 7 and 12, whereas the inversions within LGs 1, 2 and 7 are putatively under selection between migratory and nonmigratory ecotypes in the Northeast Atlantic (**b**). By grouping all individuals into migratory and nonmigratory groups (see Table 1 for details), outliers are detected within the inversions in LGs 1, 2, 7 and 12 (**c**). The plots are based on median log_10_ posterior odds (PO) values from 10 independent runs of BAYESCAN. SNPs are plotted according to linkage group and position within the linkage groups along the *x* axis as in Berg *et al.* (2016). The red line at 1 indicates ‘strong association’ according to Jeffreys (1961). The migratory group consists of Can-N_PB (Placentia Bay), Can-N_SG (Southern Gulf of St Lawrence), Ice_F (Iceland Frontal) and NEAC (Northeast Arctic cod), whereas the nonmigratory group consists of Can-S_SB (Sambro), Can-S_GM (Gulf of Maine), Can-S_BB (Browns Bank), Ice_C (Iceland Coastal) and NCC (Norwegian coastal cod). For visualization purposes, maximum log_10_ (PO) values are set to 5 (all underlying values are found in Supplementary Table S3).

Outlier analyses were performed pairwise, and identified 227 SNPs (2.8%) as candidates for divergent selection (*q*<0.01) in the Northwest Atlantic populations, 361 SNPs (4.4%) in the Northeast Atlantic, and 518 SNPs (6.3%) in all pairwise population comparisons (Supplementary Figure S6 and Supplementary Table S3). Outlier tests were also performed between the Can-N and Can-S groups, between the migratory and nonmigratory groups and between the Can-N/migratory and Can-S/nonmigratory entities, and identified 237, 319 and 365 SNPs as candidates for divergent selection, respectively (Figure 4 and Supplementary Table S3). The outlier analyses revealed four large regions potentially under selection in LGs 1, 2, 7 and 12 (Figure 4 and Supplementary Figure S6) consisting of 170, 47, 162 and 75 SNPs, respectively. The outlier regions in LGs 2 and 7 were present on both sides of the Atlantic: the outlier region in LG1 was predominantly present in Northeast Atlantic comparisons and the region in LG12 was only divergent in the Northwest Atlantic comparisons. A few additional nonlinked outliers were detected in all but two LGs (Supplementary Table S3). Of the 518 outlier SNPs, 364 are located either in or within 5 kb of a known gene, of which 196 are located in exons and 161 are nonsynonymous substitutions (Supplementary Table S3).

### LD patterns and chromosomal rearrangements

In LGs 1, 2, 7 and 12, a substantial number of SNPs were detected in high LD, forming distinct LD blocks (Supplementary Figure S7a). The strength of LD within LG1 differed between Northwest and Northeast Atlantic populations, and also between the different groups; it was greater in the Northeast Atlantic migratory populations than in the Northwest Atlantic Can-S populations (Supplementary Figure S7b). In LGs 2 and 7, the LD patterns were similar in all groups, except for the Can-N population in the Northwest Atlantic, where LD was consistently low (Supplementary Figure S7b). Similarly, in LG12, differences were observed between the Northwest and Northeast Atlantic populations, with distinctly low LD within the Can-N populations (Supplementary Figure S7b). The LD analyses also revealed smaller regions of high LD in other LGs (Supplementary Figure S7a).

**Figure 5:**
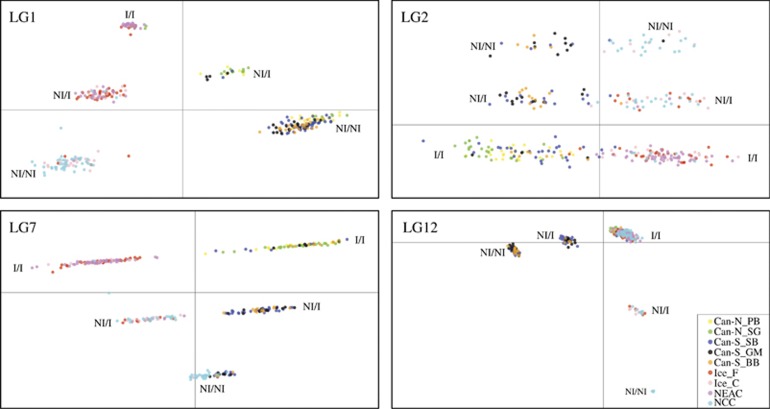
Discriminant analysis of principal components (DAPC) of the SNPs embedded within the inversions in LGs 1, 2, 7 and 12. Within all LGs a clear trimodal pattern, reflecting the inversion ‘genotypes’, is observed in addition to a broad trans-Atlantic division. Each dot represents an individual; NI/NI and I/I denote the homozygote noninverted and inverted clusters, respectively, whereas the NI/I denotes the heterozygote clusters. Inversion frequencies are listed in Supplementary Table S5. The analyses are based on n.pca=2 and n.da=2, calculated in ADEGENET. Can-N_PB, Placentia Bay; Can-N_SG, Southern Gulf of St Lawrence; Can-S_SB, Sambro; Can-S_GM, Gulf of Maine; Can-S_BB, Browns Bank; Ice_F, Iceland Frontal; Ice_C, Iceland Coastal; NEAC, Northeast Arctic cod; NCC, Norwegian coastal cod.

By using the *R* packages InveRsion and InvClust, the linked regions under selection in LGs 1, 2, 7 and 12 were identified as putative inversions, in addition to a potential inversion not under selection in LG23 (Table 2). The inversion breakpoints identified by the InveRsion package correspond well with the identified boundaries for the blocks in high LD (Supplementary Table S4). The different genotypic combinations (inversion frequencies) at LGs 1, 2, 7 and 12 (Supplementary Table S5) contribute to the observed population and ecotype divergence in addition to a trans-Atlantic difference (Table 2 and Figure 5), whereas the LG23 region primarily shows a trans-Atlantic difference. *F*_ST_ values within the homozygote inverted and noninverted variants were calculated for all four divergent regions across the Atlantic Ocean (Table 2), showing large increased diversity in the noninverted variant in LG1 and LG12 and in the inverted variant in LG7, relative to the genome-wide neutral divergence. These results are consistent with the spatial distribution visualized in Figure 5. In addition, low genomic divergence, reflected by heterozygosity (Supplementary Figure S2) and nucleotide diversity (*π*, Table 2 and Supplementary Figure S3) was observed for the migratory and Can-S groups within the divergent regions in LGs 1, 2, 7 and 12.

The inversions vary in size and number of genes: LG1, at least 18.5 Mb and 785 genes; LG2, ∼6 Mb and 293 genes; LG7, at least 10 Mb and 324 genes; LG12, ∼13 Mb and 419 genes; LG23, >3.5 Mb and 97 genes (Supplementary Table S6). Combined, the divergent regions in LGs 1, 2, 7, 12 and 23 are >50 Mb (≈6% of the genome) and contain >1900 genes (Supplementary Table S6).

## Discussion

### Polymorphic inversions

Ever since seasonal changes in inversion frequencies were observed in *Drosophila* (Dobzhansky, 1943), the effects of reduced recombination rates within inversions have been linked to adaptation with gene flow, and investigations have shown that sympatric species exhibit more differences caused by inversions than allopatric species (see, for example, Rieseberg, 2001; Noor *et al.*, 2001; Ayala and Coluzzi, 2005; Kandul *et al.*, 2007). Recent research on tropical reef fishes (Martinez *et al.*, 2015) and Estrildid finches (Hooper and Price, 2015) indicate a quicker fixation of inversions in lineages with higher dispersal potential and gene flow, consistent with a theory where gene flow favours diversification of chromosomal rearrangements caused by locally adapted loci (Kirkpatrick and Barton, 2006). In line with these findings, we observe generally low genome-wide divergence interspersed with highly divergent regions (Figure 4) among the investigated Atlantic cod populations, where gene flow could potentially be high because of few physical barriers. In an adaptation with gene flow scenario where different ecotypes are maintained in close proximity and potentially interbreeding, such as in the case of NEAC/NCC and Ice-F/Ice-C, inversion polymorphism (effectively acting as supergenes) could be an important factor in upholding the ecotype diversity. This has been shown in *Heliconius* butterflies, where supergenes controlling wing mimicry have been attributed to a series of inversions that suppress recombination (Joron *et al.*, 2011; Jones, Salazar, *et al.*, 2012). Chromosomal rearrangements have also been associated with behavioural or ecotype differences in other species such as white-throated sparrow (*Zonotrichia albicollis*) (Zinzow-Kramer *et al.*, 2015), rainbow trout (*Oncorhynchus mykiss*) (Pearse *et al.*, 2014) fire ant (*Solenopsis invicta*) (Wang *et al.*, 2013), stickleback (*Gasterosteus aculeatus*) (Jones, Grabherr, *et al.*, 2012) and *Anopheles* mosquitoes (Love *et al.*, 2016). Recently, a haplotype block and a potential inversion associated with different spawning times have been identified in herring (*Clupea harengus*) across the Atlantic Ocean (Lamichhaney *et al.*, 2017).

In our data set, we observe four distinct large genomic regions of divergence where SNPs are in persistently high LD with each other. These regions are likely chromosomal inversions over several Mb in size (Berg *et al.*, 2016; Kirubakaran *et al.*, 2016) that segregate as biallelic loci within populations. As such, inversions that contain multiple genes involving a certain set of phenotypic traits could be responsible for maintaining vital polymorphisms within the Atlantic cod genome. Alternatively, the observed pattern could also result from secondary contact between previously diverged populations where the inversions form endogenous incompatibilities protected from recombination. In such a scenario, selection would not cause or maintain the inversions *per se*, but secondary contact could result in genomic homogenization across the genome except for the inverted regions. If so, the inversions would maintain the differentiation by coupling to loci associated with the divergence that is consistent with the coupling hypothesis (Bierne *et al.*, 2011). Nonetheless, we find this explanation less likely as this hypothesis implies fixation of alternative alleles due to genetic incompatibilities, whereas we observe high frequencies of heterozygous individuals at all the inversions. In addition, a large number of F1 hybrids between the Can-N and Can-S groups have been observed (Bradbury *et al.*, 2014) and crosses of breeding stocks of NEAC and NCC are routinely made (Bangera *et al.*, 2015), suggesting a lack of lethal genetic incompatibilities between the potential inversion types.

Regardless of the cause of the selective advantage of the inversions, these islands comprise ‘fixed’ entities that behave like biallelic loci and that appear to resist introgression even though interbreeding in areas of sympatry have been observed (Bradbury *et al.*, 2014). Our results are consistent with the hypothesis that broad-acting selective agents target numerous biological functions. This combined with relatively low level of pairwise genetic divergence throughout the rest of the genome within the Northwest and Northeast Atlantic cod populations suggests that the divergence within the rearrangements is indicative of adaptive divergence. This has also been shown in perennial and annual ecotypes of monkeyflower that differ significantly within an inversion, while high gene flow homogenizes the collinear parts of the genome (Twyford and Friedman, 2015).

### Trans-Atlantic genomic divergence

Cod have been extant on both sides of the Atlantic for >100 000 years (Bigg *et al.*, 2008; Carr and Marshall, 2008). Consistent with the expectations of allopatric population differentiation, a distinct separation is observed across the breadth of the species’ range (Figure 3a), concordant with both mitochondrial DNA data (Árnason, 2004; Bigg *et al.*, 2008; Carr and Marshall, 2008) and nuclear DNA data (Bentzen *et al.*, 1996; Pogson, 2001; O'Leary *et al.*, 2007). Demographic processes appear to explain most of the genomic differentiation between the Northwest and Northeast Atlantic populations, although SNPs within a presumptive inversion in LG23 exhibit elevated *F*_ST_ values relative to the genomic average. The putative inversion in LG23 contains at least 97 genes, where two outlier SNPs were detected in a voltage-dependent calcium channel gene (*CACNA1S*) known to be expressed in early-stage embryos of *Danio rerio* (Sanhueza *et al.*, 2009). Alternative alleles within this region are nearly fixed between the Northwest and Northeast Atlantic populations (Table 2), with no differentiation within these locations. Low heterozygosity and low nucleotide diversity within the presumed inversion in the Northwest Atlantic populations indicate that this might be the derived variant. The origin of the inversion is likely to pre-date the trans-Atlantic split as both variants have a trans-Atlantic presence (although at low respective frequencies). In addition, there are two distinct population clusters within both the Northwest and the Northeast Atlantic that cannot be attributed to trans-Atlantic divergence. Here, the main genetic differences are attributed to inversions within LGs 1, 2, 7 and 12.

### Genomic divergence within the Northeast Atlantic

Even though a distinction between Coastal and Frontal ecotypes in Icelandic waters has been investigated recently (see, for example, Pampoulie *et al.*, 2008; Grabowski *et al.*, 2011; Pampoulie *et al.*, 2015), the genomic basis for these differences has not yet been examined in detail. However, *F*_ST_ patterns in Icelandic waters between coastal and deep-water populations that were not characterized with DSTs (that is, not real migratory vs nonmigratory ecotypes) have been described as reflecting a differentiation similar to that reported between NEAC and NCC populations (Hemmer-Hansen *et al.*, 2013). To date, true Coastal and Frontal ecotypes can only be distinguished by DST profiles (see Pálsson and Thorsteinsson, 2003; Thorsteinsson *et al.*, 2012), although they exhibit different *Pan* I locus genotypes and differ at the *RH1* opsin gene (Pampoulie *et al.*, 2008, 2015), with both genes residing within the LG1 inversion. Our data show that most of the genomic differentiation between the Coastal and Frontal ecotypes (as defined by DSTs) can be attributed to the LG1 inversion, but that smaller *F*_ST_ differences are also observed at LGs 2 and 7. We do not observe any significant neutral divergence (*F*_ST_=0.0002) between Frontal and Coastal ecotypes, whereas significant nonneutral divergence (mainly within LGs 1, 2 and 7) are observed (*F*_ST_=0.0547). Both the Frontal and the Coastal ecotypes are inshore cod that spawn at the same spawning grounds and at the same time. The observed pattern with no neutral divergence, and significant nonneutral divergence, is consistent with a divergent selection hypothesis where individuals potentially interbreed at the spawning grounds followed by *de novo* selection, discriminating the two ecotypes.

Both the Frontal and Coastal ecotypes cluster close to the NEAC population and away from the NCC population (Figure 3d), as indicated by the low neutral divergence between both Coastal and Frontal ecotypes relative to the NEAC (0.0025 and 0.0026) and NCC (0.0062 and 0.0068). Non-neutral divergence between Frontal and Coastal ecotypes are higher than the divergence between Frontal and NEAC (0.0349), but lower than the observed divergence between Frontal and NCC (0.2507) and between Coastal and both NEAC and NCC (0.1610 and 0.1142). Hence, the two Icelandic ecotypes may be derived from NEAC, where local adaptations are forming migratory and nonmigratory ecotypes based on standing genetic variation in the putative inversions in the NEAC genome. As such, the differentiation that we observe between Ice_C and Ice_F, which has been grouped according to behaviour (based on DST tags), probably reflect ecotype divergence in a similar way.

### Genomic divergence within the Northwest Atlantic

Within the Northwest Atlantic, the populations cluster into a Can-N and a Can-S group that are known to occupy different thermal regimes (Hutchings *et al.*, 2007; Bradbury *et al.*, 2010, 2013). The northern populations Can-N_PB (Placentia Bay) and Can-N_SG (Southern Gulf of St Lawrence) belong to the ‘cold’ group, the southern populations Can-S_GM (Gulf of Maine) and Can-S_BB (Browns Bank) belong to the ‘warm’ group and the Can-S_SB (Sambro) is located at the transition between the two groups. For the northern populations, tagging experiments show that large individuals of the Can-N_PB population perform relatively long annual migrations (>100 km and up to 500 km) (Lawson and Rose, 2000) with comparatively precise homing to their natal area (Robichaud and Rose, 2011), and the Can-N_SG is known to exhibit even longer annual migration patterns of >225 km for juveniles and up to 650 km for adult fish (Hanson, 1996). In the southern populations, the Can-S_GM population performs limited annual migration within the Gulf of Maine (<65 km) (Ruzzante *et al.*, 1998) and the Can-S_BB are described as being resident to the bank (Zemeckis *et al.*, 2014), where eggs and larvae are likely to be retained by gyres around the bank. Little is currently known about the migration patterns of the Can-S_SB population, but other populations on the Scotian shelf show limited migration patterns (Ruzzante *et al.*, 1998). The northern populations Can-N_PB and the Can-N_SG spawn in the summer months, the southern populations Can-S_GM and Can-S_BB spawn in late winter/early spring and the Can-S_SB population spawns in late fall (Table 1). As such, there are several notable differences between the Can-N and Can-S groups that are not easily disentangled, such as temperature, spawning time and migratory behaviour. It is clear, however, that the divergence between the Can-N and the Can-S ‘ecotypes’ is not only driven mainly by differences in the inversions in LGs 2, 7 and 12 (reflected by both inversion frequencies, *F*_ST_ values and outlier patterns), but also to some extent by differences in the LG1 inversion. This is consistent with results reported by Bradbury *et al.* (2010) and Hemmer-Hansen *et al.* (2013) who identified outlier regions primarily within LGs 2, 7 and 12 among Northwest Atlantic populations, but did not attribute this divergence to inversions or to ecotype differentiation. Lately, significant population differentiation has also been observed between spawning groups within the Gulf of Maine and between Georges bank and Gulf of Maine at three large genomic regions in LGs 2, 7 and 12 and increased *F*_ST_ values was observed between spring and winter spawning populations within the LG2 region (Barney *et al.*, 2017).

### Population and ecotype differentiation within the divergent regions

The inversion in LG1 is involved in ecotype divergence of Northeast Atlantic populations (Hemmer-Hansen *et al.*, 2013; Berg *et al.*, 2016; Kirubakaran *et al.*, 2016) but shows a less pronounced divergence between Can-N and Can-S in the Northwest Atlantic populations. However, the presumably ancestral (NI/NI) inversion ‘genotype’, found predominantly in the nonmigratory ecotype in the Northeast Atlantic, is found at similarly high frequencies in the Can-S populations. The NI/NI and the I/I variants are highly divergent across the Atlantic (*F*_ST_=0.237 and 0.159, respectively), relative to the neutral trans-Atlantic divergence (*F*_ST_=0.081). This suggests local selection pressures acting differently on both of the variant on each side of the Atlantic, but less so for the I/I variant that is associated with migratory behaviour in the Northeast Atlantic. The fact that we detect a similar trans-Atlantic pattern at the LG1 inversion is consistent with Bradbury *et al.* (2010) that northern samples from the Northwest Atlantic (Davis Strait) and samples from the Barents Sea (presumably NEAC) were not significantly divergent in this region.

SNPs within the presumed inversions in LGs 2 and 7 have previously been linked to temperature (Bradbury *et al.*, 2010), salinity and oxygen levels in the Baltic Sea (Berg *et al.*, 2015), and ecotype divergence of Northeast Atlantic populations (Berg *et al.*, 2016), and are known to be divergent in a wide range of cod populations across the Atlantic (Bradbury *et al.*, 2010; Hemmer-Hansen *et al.*, 2013). Interestingly, within both of these LGs, the Can-N populations are fixed for the I/I variant that is nearly fixed in the migratory ecotypes in the Northeast Atlantic. Notably, there are clear *F*_ST_ differences between the Ice_C and Ice_F ecotypes but these differences are not strong enough to manifest as potential outliers in the outlier tests. Intriguingly, we also observe elevated *F*_ST_ difference in LG7 between the Ice_C (nonmigratory) and NCC populations that may seem inconsistent with the proposed association with migratory behaviour. However, this might reflect the fact that the Ice_C ecotype presumably has a NEAC origin that consists predominantly of the I/I variant. Hence, the standing genomic divergence may not contain sufficient variation-needed for adaptation. Alternatively, the selection pressure on this genomic region might not be strong enough to cause significant differentiation within the selection timeframe.

The presumably inverted genomic region in LG12 has recently been used to discriminate between two Atlantic cod stocks inhabiting the Norwegian Skagerrak coast (Sodeland *et al.*, 2016), and SNPs within this region have been linked to temperature in two separate studies (Bradbury *et al.*, 2010; Berg *et al.*, 2015). This inversion is fixed or nearly fixed for the I/I variant in all populations in our study except for the three Can-S populations that are highly polymorphic. The frequencies of the inversion in LG12 does not differ significantly between the NEAC and NCC populations (Berg *et al.*, 2016), indicating that this inversion may not distinguish migratory from nonmigratory ecotypes *per se.* Hence, the observed differences between Can-N and Can-S may reflect adaptation to different thermal regimes. Interestingly, the trans-Atlantic divergence at the NI/NI variant (*F*_ST_=0.736) is high relative to the I/I variant (*F*_ST_=0.076) and the neutral trans-Atlantic divergence (*F*_ST_=0.081) that may indicate local selection pressure acting differently on the NI variant in Northeast and Northwest Atlantic.

Identifying the actual targets of selection within inversions or other tightly linked genomic regions is challenging, as recombinations are reduced within inversions causing difficulties in distinguishing true targets of selection from linked false positive signals. We have postulated that both of the Icelandic ecotypes are derived from NEAC. Hence, if the divergence among the Icelandic samples reflects true ecotype divergence, constituted by *de novo* selection in each generation, the resulting genomic divergence will be based on the standing genetic variation present in the NEAC genome. In NEAC (and Icelandic populations), the I/I variant is almost fixed in LGs 2 and 7 (frequency: 0.98 and 0.94). As a result, almost all variation within the putative LG 2 and 7 inversions will be collinear, allowing for normal recombination to take place. As such, the genomic divergence between Coastal and Frontal ecotypes within these regions may provide valuable insight into the actual targets of selection, because the normal limitations associated with highly linked genomic regions here are omitted. The highest *F*_ST_ values between Coastal and Frontal ecotypes are found in a SNP close to Synaptotagmin (*SYT3*) in LG2 (*F*_ST_=0.1152) and in two SNPs (*F*_ST_=0.1240 and 0.1222) associated with Tyrosinase (*TYR*), one of which is nonsynonymous in LG7. *SYT3* is known to show different expression patterns in resident ‘sneaker’ individuals of Atlantic Salmon (*Salmo salar*) relative to normal migratory individuals (Aubin-Horth *et al.*, 2005), supporting a potential role in behavioural ecotype divergence. *TYR* is considered as a clock-controlling gene (Moraes *et al.*, 2014), known to control circadian rhythm of several physiological and behavioural processes (Reppert and Weaver, 2001, 2002). *TYR* is also involved in regulation of melanin production that influences both skin and retinal pigmentation potentially relevant to vision, depth adaptation and hence vertical migration in fish. This is an interesting finding, as rhodopsin (*RH1*)—a gene known to mediate dim light vision—is strongly divergent between the two Icelandic ecotypes, indicating an involvement of visual systems in local adaptation of Atlantic cod (Pampoulie *et al.*, 2015).

Combined, our results suggest that: (1) all of the inversion events occurred before the split between Northeast and Northwest Atlantic cod populations, ∼100 000 years ago; (2) the nonmigratory/Can-S group is always dominated by the ancestral collinear inversion genotype (NI/NI) containing the highest nucleotide diversity; (3) nonmigratory behaviour in the Northeast Atlantic appears to be ancestral to migratory behaviour; and (4) inshore Icelandic ecotypes have a presumed NEAC origin. As such, we provide fundamental insight into the evolution of distinct morphs and ecotypes of Atlantic cod with different life-history strategies across the trans-Atlantic barrier. Overall, the data indicate a central role for a few distinct large genomic regions, presumably inversions. The genomic content of these regions are targets of selection, likely to be involved in generating and maintaining adaptive divergence and population differentiation among Atlantic cod throughout its distribution range.

## Data archiving

All SNPs are referred to by their ss# or rs# available in dbSNP at www.ncbi.nlm.nih.gov/SNP/. All individual genotype data are available from the Dryad data repository (http://dx.doi.org/10.5061/dryad.b20ps).

## Figures and Tables

**Table 1:** Sample details of the Atlantic cod samples included in this study and basic population genetic parameters

*Sampling ID*	*Group*	*Sampling time*	*Spawning time*	*Lat.*	*Long.*	*Condition*	*Ind. call no. >0.95*	*Avg. call rate*	*No. of polymorphic loci*	H_*o*_ *(s.d.)*	H_*e*_ *(s.d.)*
Can-N_PB	Can-N	July 2007	April–July	N47.15	W54.15	Juveniles^a^	24	0.989	7351	0.308 (0.172)	0.309 (0.156)
Can-N_SG	Can-N	May 2001	May–June	N46.13	W61.39	Adults^b^	24	0.990	6933	0.325 (0.177)	0.316 (0.153)
Can-S_SB	Can-S	Dec 2010	Nov–Dec	N44.27	W63.36	Adults^b^	48	0.994	7876	0.308 (0.167)	0.309 (0.157)
Can-S_GM	Can-S	July 2009	Feb–April	N43.16	W67.46	Adults	24	0.994	7657	0.322 (0.171)	0.320 (0.153)
Can-S_BB	Can-S	July 2009	Mar–April	N42.35	W65.50	Adults	24	0.994	7651	0.314 (0.168)	0.319 (0.154)
Ice_F	Migratory		Mar–May			Adults^b^	39	0.994	8064	0.358 (0.152)	0.357 (0.138)
Ice_C	Nonmigratory		Mar–May			Adults^b^	39	0.994	8053	0.363 (0.146)	0.365 (0.133)
NEAC	Migratory	Mar. 2011	Mar–May	N68.19	E13.30	Adults^b^	50	0.998	8039	0.357 (0.147)	0.356 (0.137)
NCC	Nonmigratory	Jun/Jul 2011	Mar–May	N68.04	E13.41	Adults/juv.	44	0.996	8126	0.366 (0.143)	0.367 (0.129)

Abbreviations: Avg., average; Can-N_PB, Placentia Bay; Can-N_SG, Southern Gulf of St Lawrence; Can-S_SB, Sambro; Can-S_GM, Gulf of Maine; Can-S_BB, Browns Bank; Ice_F, Iceland Frontal; Ice_C, Iceland Coastal; Ind., individual; Lat., latitude; Long., longitude; NEAC, Northeast Arctic cod; NCC, Norwegian coastal cod.

The Icelandic samples were collected at several locations in the waters around Iceland and later categorized as Frontal or Coastal, based on Data Storage Tag profiling. See Figure 1 in Thorsteinsson *et al.* (2012) for a detailed view of the Icelandic sampling localities.

Estimates of observed (*H*o) and expected heterozygosity (*H*e) were calculated using ARLEQUIN. Latitude and longitude values are given in degrees and minutes and the coordinates for Can-N_PB, Can-S_GM and Can-S_BB are approximations.

a Produced by wild-caught adults in spawning condition.

b In spawning condition.

**Table 2:** Chromosomal rearrangements in Atlantic cod, their contribution to population genetic structure reflected by the frequencies of the inversions and nucleotide diversity between and within groups of populations based on SNPs within the inversions

*LG*	*Inversion breakpoint*	*Inversion frequencies*												*Nucleotide diversity between groups (*D_XY_)		*Nucleotide diversity within groups (*π)				F_*ST*_ *values*	
	*(SNP no.)*	*Northwest Atlantic*			*Northeast Atlantic*			*Mig-Can-N*^a^			*N.Mig-Can-S*^a^			*Northwest/ Northeast Atlantic*	*Mig-Can-N/N.Mig-Can-S*	*Northwest Atlantic*	*Northeast Atlantic*	*Mig- Can-N*^a^	*N.Mig*-*Can-S*^a^	*Trans-Atlantic*	
		*NI*	*NI/I*	*I*	*NI*	*NI/I*	*I*	*NI*	*NI/I*	*I*	*NI*	*NI/I*	*I*							*NI/NI*	*I/I*
1	135–415	0.81	0.16	0.03	0.40	0.39	0.21	0.23	0.50	0.27	0.86	0.12	0.02	0.36	0.36	0.24	0.36	0.29	0.33	0.237	0.159
2	748–832	0.14	0.26	0.60	0.15	0.24	0.61	0.00	0.09	0.91	0.25	0.37	0.37	0.33	0.33	0.29	0.32	0.22	0.36	0.077	0.106
7	2533–2717	0.15	0.38	0.48	0.15	0.29	0.56	0.00	0.11	0.89	0.26	0.50	0.24	0.40	0.45	0.37	0.39	0.19	0.42	0.128	0.209
12	4248–4443	0.29	0.28	0.43	0.02	0.10	0.88	0.00	0.04	0.96	0.25	0.28	046	0.29	0.28	0.26	0.23	0.18	0.27	0.736	0.076
23	7862–7916	0.01	0.06	0.93	0.88	0.12	0.01	0.58	0.07	0.34	0.41	0.10	0.49	0.37	0.32	0.17	0.32	0.32	0.31		

Abbreviations: I, inverted; LG, linkage group; Mig, migratory; NI, noninverted; N.Mig, nonmigratory; SNP, single-nucleotide polymorphism.

All rearrangements are detected with the InveRsion package (Cáceres *et al.*, 2012) and inversion breakpoints are the identified left (min) and right (max) values. Rearrangement frequencies for all populations separately are given in Supplementary Table S5.

SNP number (no.) corresponds to the SNP numbering in Supplementary Table S3. NI is the presumably non-inverted (ancestral) homozygote state of the inversion, and I is the presumably inverted (derived) homozygote state of the inversion and NI/I is the heterozygote state of the inversion (see main text for details).

Northwest Atlantic: Can-N_PB, Placentia Bay; Can-N_SG, Southern Gulf of St Lawrence; Can-S_SB, Sambro; Can-S_GM, Gulf of Maine; Can-S_BB, Browns Bank. Northeast Atlantic: Ice_F, Iceland Frontal; Ice_C, Iceland Coastal; NEAC, Northeast Arctic cod; NCC, Norwegian coastal cod.

a These groups consist of individuals from both sides of the Atlantic Ocean, resulting in the observed heterozygote deficiency in LG23 because of almost fixed trans-Atlantic frequency differences.
