# Supplementary material for: Trans-oceanic genomic divergence of Atlantic cod ecotypes is associated with large inversions
Source: Heredity (Edinb). 2017 Sep 20;119(6):418–28. doi: 10.1038/hdy.2017.54 (PMC5677996; doi:10.1038/hdy.2017.54)
Supplement: Supplementary Figures [file hdy201754x1.pdf]

Supplementary Figures

Berg et al. 2017 " Inversions play a key role in ecotype divergence of Atlantic cod across the Atlantic Ocean"

Northwest Atlantic F_{ST} pattern

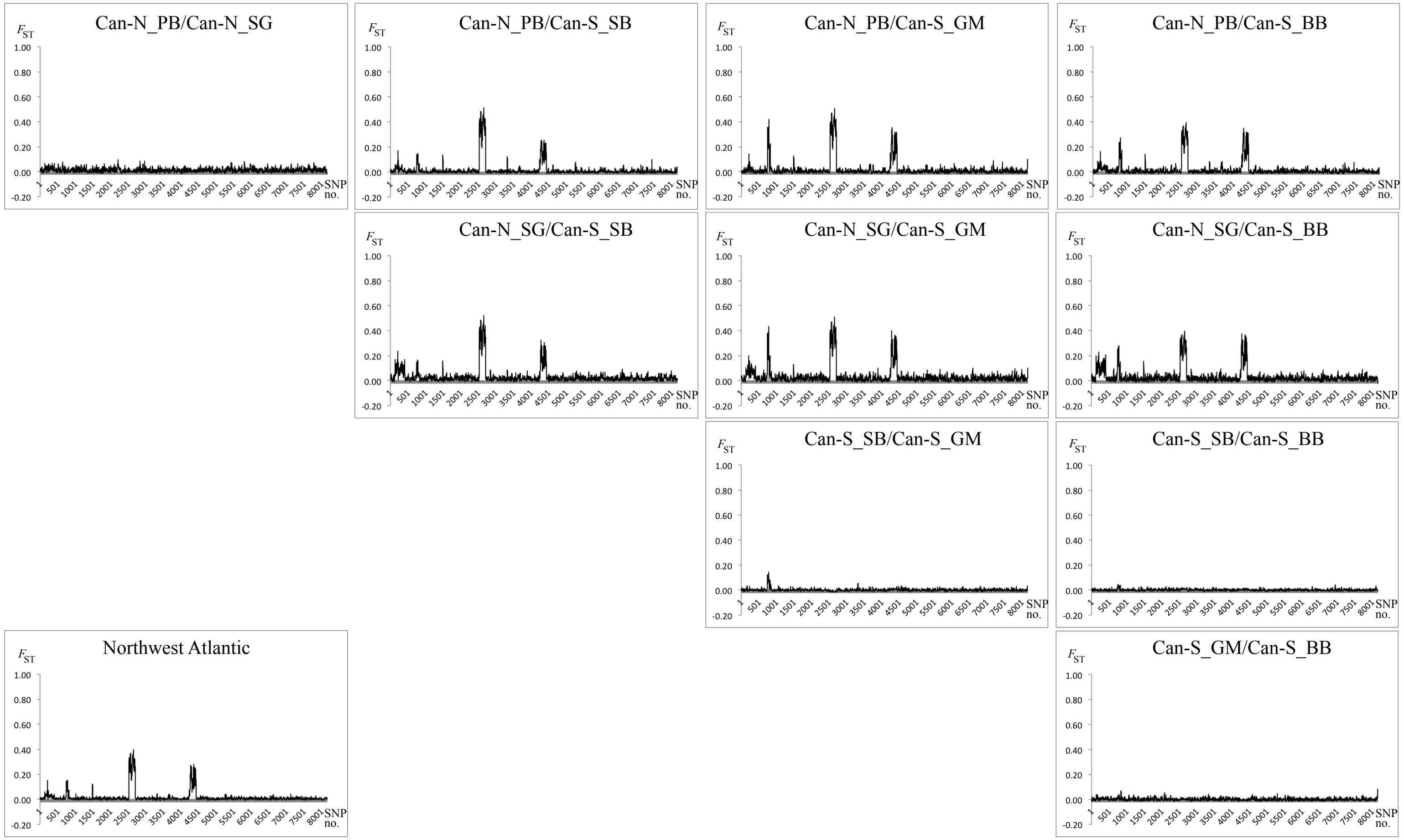

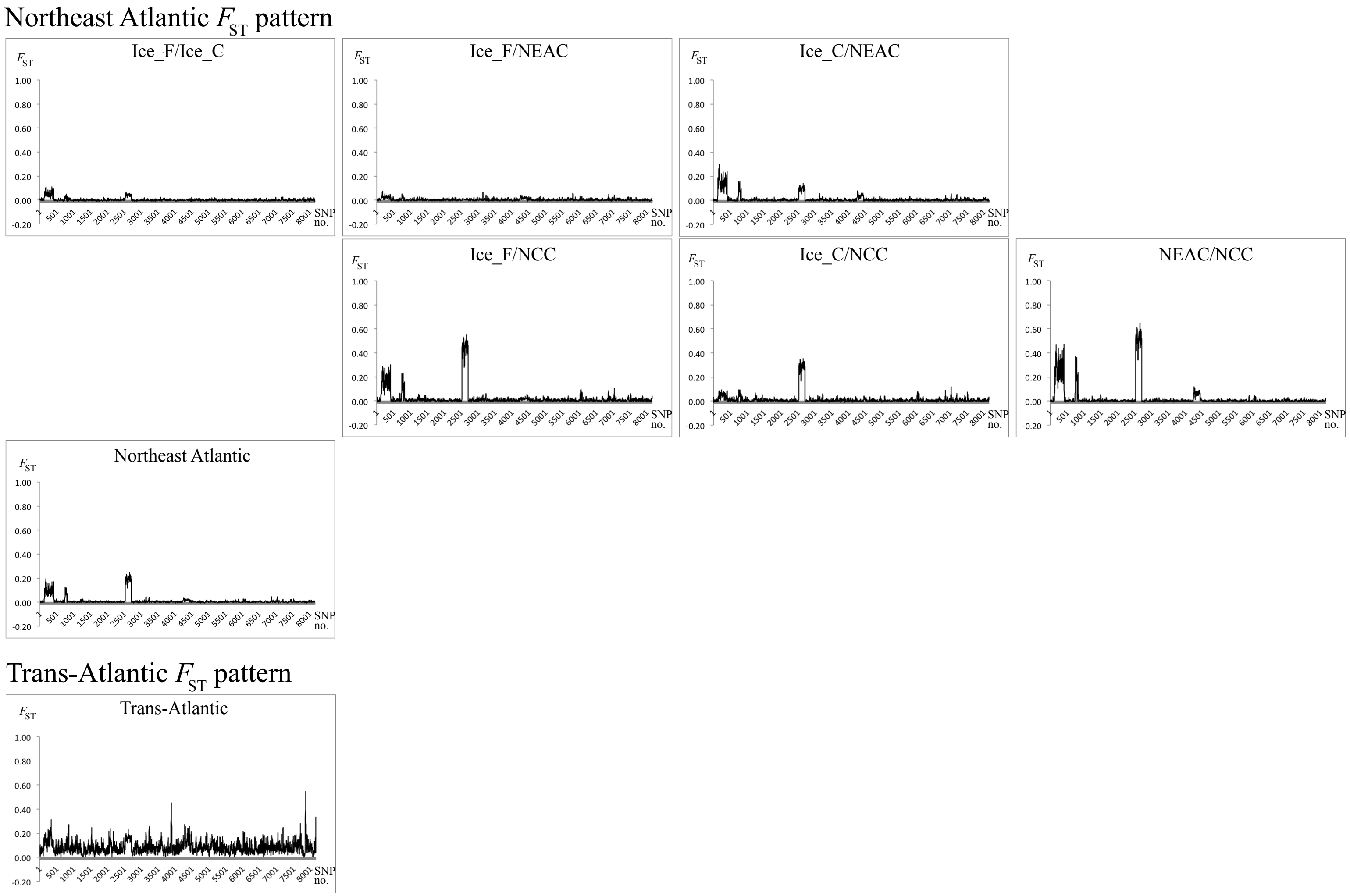

Supplementary Fig. S1. **Locus specific F_{ST} values in Atlantic cod for all pairwise population comparisons in the Northwest and Northeast Atlantic.** The observed F_{ST} pattern shows distinct genomic regions with elevated F_{ST} values. In the Northwest Atlantic, the majority of elevated F_{ST} values is observed between the Can-N and the Can-S groups while in the Northeast Atlantic the majority of elevated F_{ST} values is observed between the migratory and non-migratory groups. SNPs are ordered according to linkage group and position within the linkage groups along the X-axis as in Berg *et al.* (2016). Can-N (Can-N_PB = Placentia Bay, Can-N_SG = Southern Gulf of St. Lawrence), Can-S (Can-S_SB = Sambro, Can-S_GM = Gulf of Maine, Can-S_BB = Browns Bank), migratory (Ice_F = Iceland Frontal, NEAC = Northeast Arctic cod), non-migratory (Ice_C = Iceland Coastal, NCC = Norwegian coastal cod).

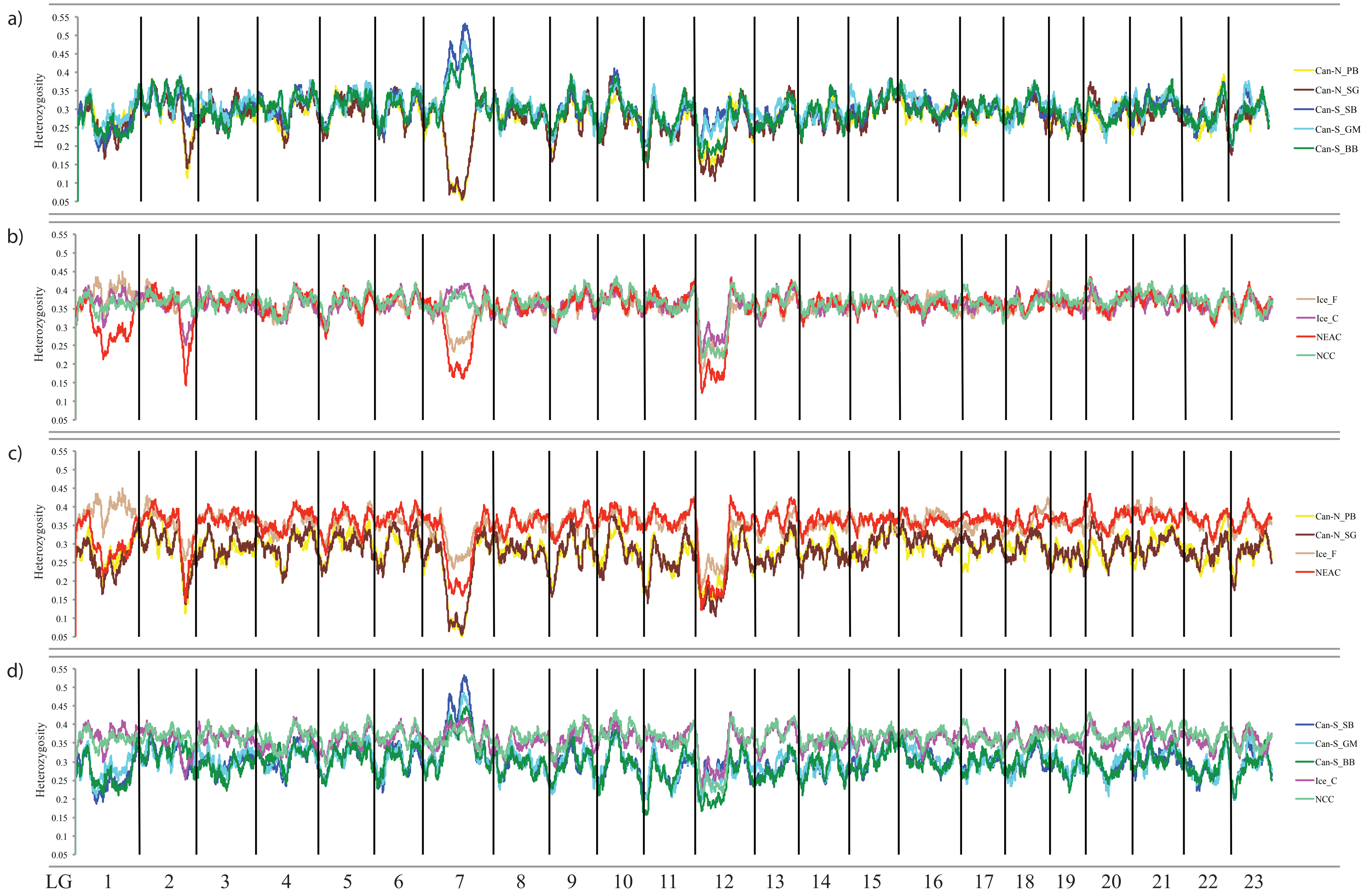

Supplementary Fig. S2. **Heterozygosity level across all linkage groups in Atlantic cod.** The observed heterozygosity levels in (**a**) the Northwest Atlantic populations, (**b**) the Northeast Atlantic populations, (**c**) Can-N and migratory populations and (**d**) Can-S and non-migratory populations. Combined, the observed heterozygosity pattern shows four distinct regions of the genome with distinctly different heterozygosity patterns. SNPs are ordered according to linkage group and position within the linkage groups along the X-axis as in Berg *et al.* (2016). Can-N (Can-N_PB = Placentia Bay, Can-N_SG = Southern Gulf of St. Lawrence), Can-S (Can-S_SB = Sambro, Can-S_GM = Gulf of Maine, Can-S_BB = Browns Bank), migratory (Ice_F = Iceland Frontal, NEAC = Northeast Arctic cod), non-migratory (Ice_C = Iceland Coastal, NCC = Norwegian coastal cod).

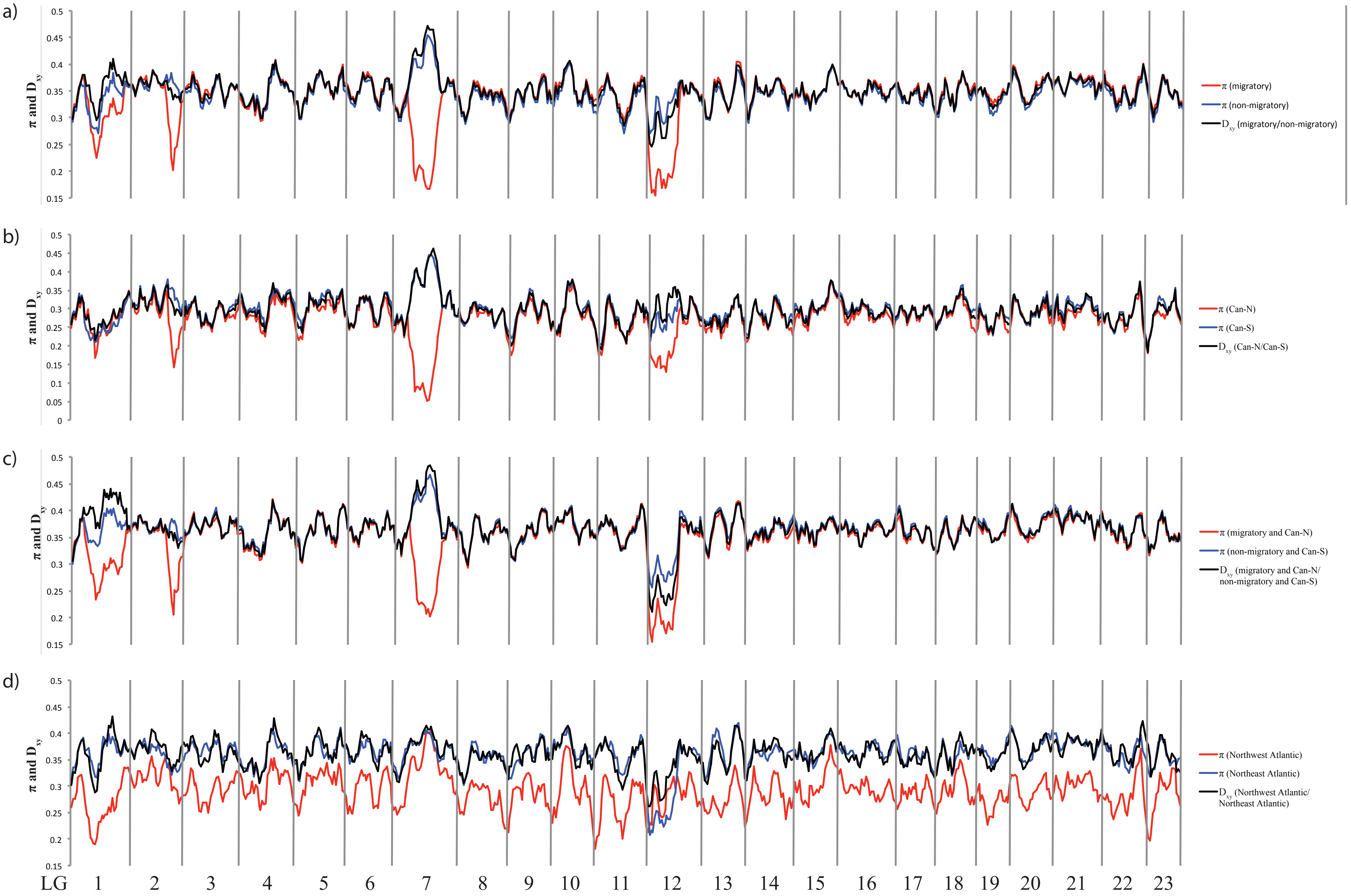

Supplementary Fig. S3. **Nucleotide diversity (π) and nucleotide divergence (D_{XY}) in Atlantic cod.** The calculated π within and D_{XY} between: (**a**) the migratory and non-migratory groups in the Northeast Atlantic, (**b**) the Can-N and Can-S groups in the Northwest Atlantic, (**c**) the migratory and Can-N groups combined vs. the non-migratory and Can-S group combined and (**d**) the Northwest Atlantic populations vs. the Northeast Atlantic populations. Calculations were performed using a sliding windows approach with a 50-SNP window and 10 SNPs per iteration in DnaSP 5.1064. SNPs are ordered according to linkage group and position within the linkage groups along the X-axis as in Berg *et al*. (2016). Can-N (Can-N_PB = Placentia Bay, Can-N_SG = Southern Gulf of St. Lawrence), Can-S (Can-S_SB = Sambro, Can-S_GM = Gulf of Maine, Can-S_BB = Browns Bank), migratory (Ice_F = Iceland Frontal, NEAC = Northeast Arctic cod), non-migratory (Ice_C = Iceland Coastal, NCC = Norwegian coastal cod).

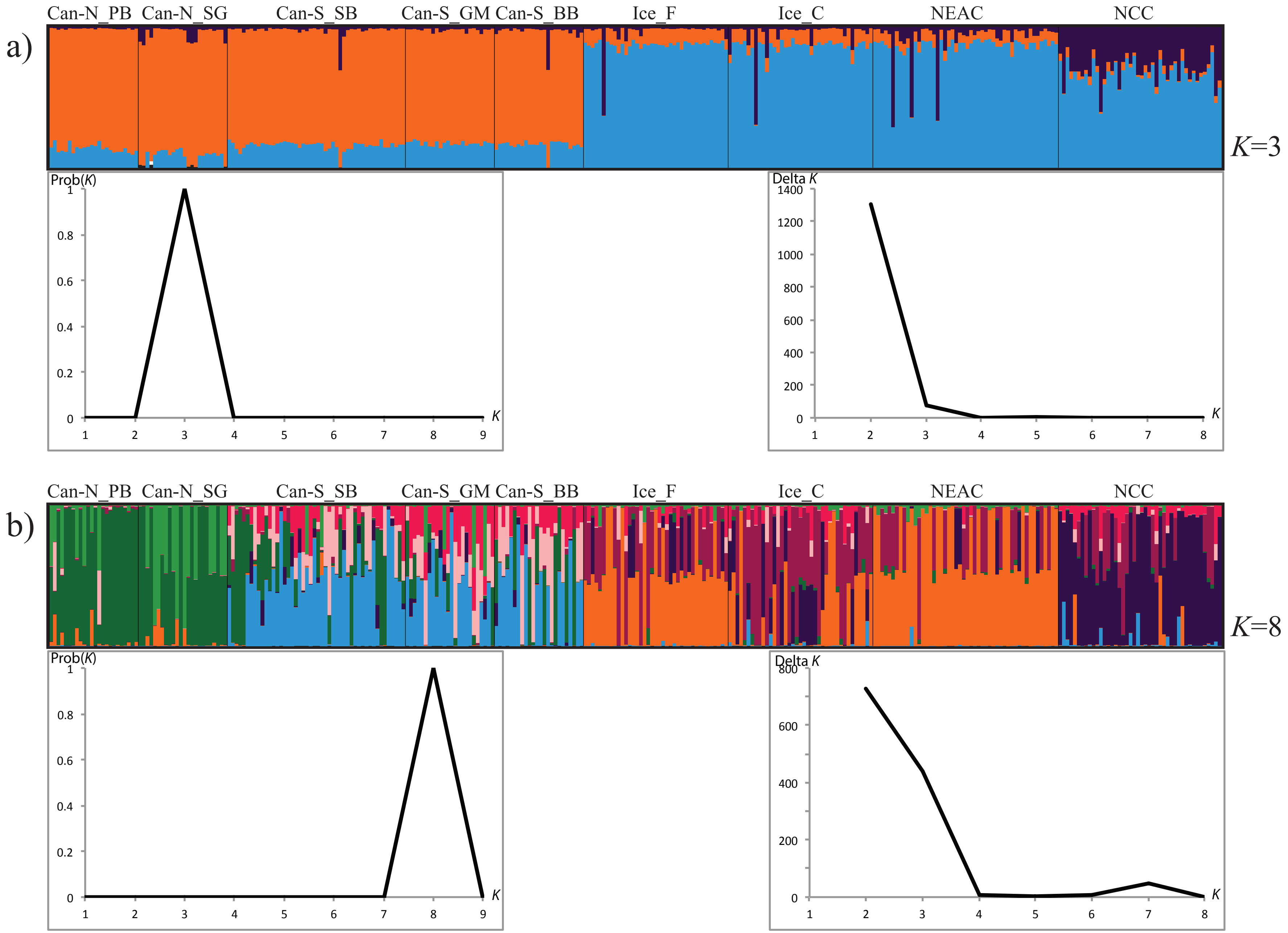

Supplementary Fig. S4. **Structure plots of the assignment probabilities in Atlantic cod.** **(a)** STRUCTURE plot of the neutral dataset (7,075 tag-SNPs) at *K*=3 together with a plot showing the most likely *K* value and Delta *K* and (**b**) the outlier dataset (325 tag-SNPs) at *K*=8 together with a plot showing the most likely *K* value and Delta *K*. For each STRUCTURE plot, a bar represents each individual sample and the plots are based on the combined results from 10 independent STRUCTURE runs. Can-N_PB = Placentia Bay, Can-N_SG = Southern Gulf of St. Lawrence, Can-S_SB = Sambro, Can-S_GM = Gulf of Maine, Can-S_BB = Browns Bank, Ice_F = Iceland Frontal, Ice_C = Iceland Coastal, NEAC = Northeast Arctic cod, NCC = Norwegian coastal cod.

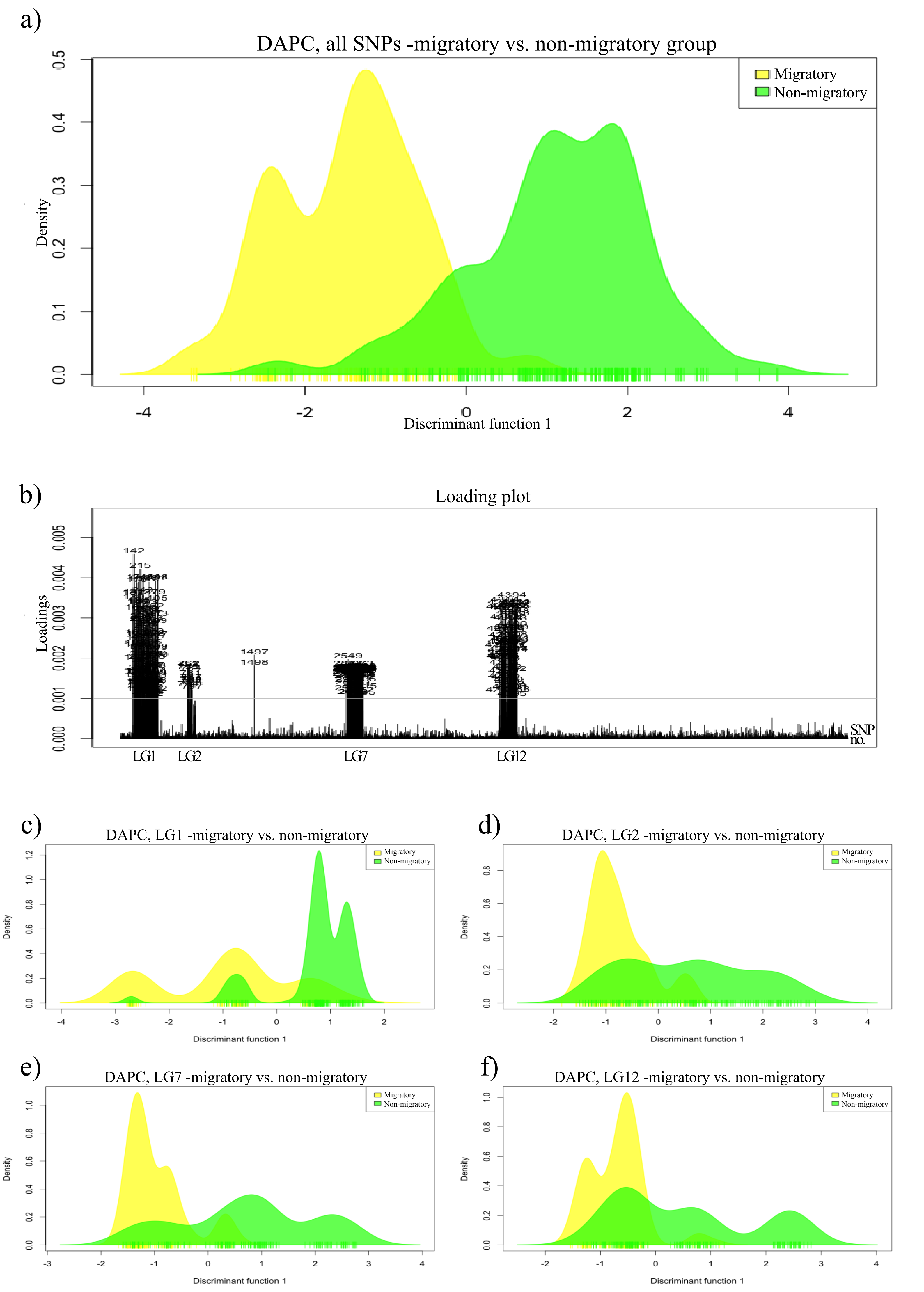

a)
DAPC, all SNPs -migratory vs. non-migratory group
Migratory
Non-migratory
Density
Discriminant function 1
b)
Loading plot
Loadings
SNP no.
LG1
LG2
LG7
LG12
c)
DAPC, LG1 -migratory vs. non-migratory
d)
DAPC, LG2 -migratory vs. non-migratory
e)
DAPC, LG7 -migratory vs. non-migratory
f)
DAPC, LG12 -migratory vs. non-migratory

g)

DAPC, all LGs separately-migratory vs. non-migratory

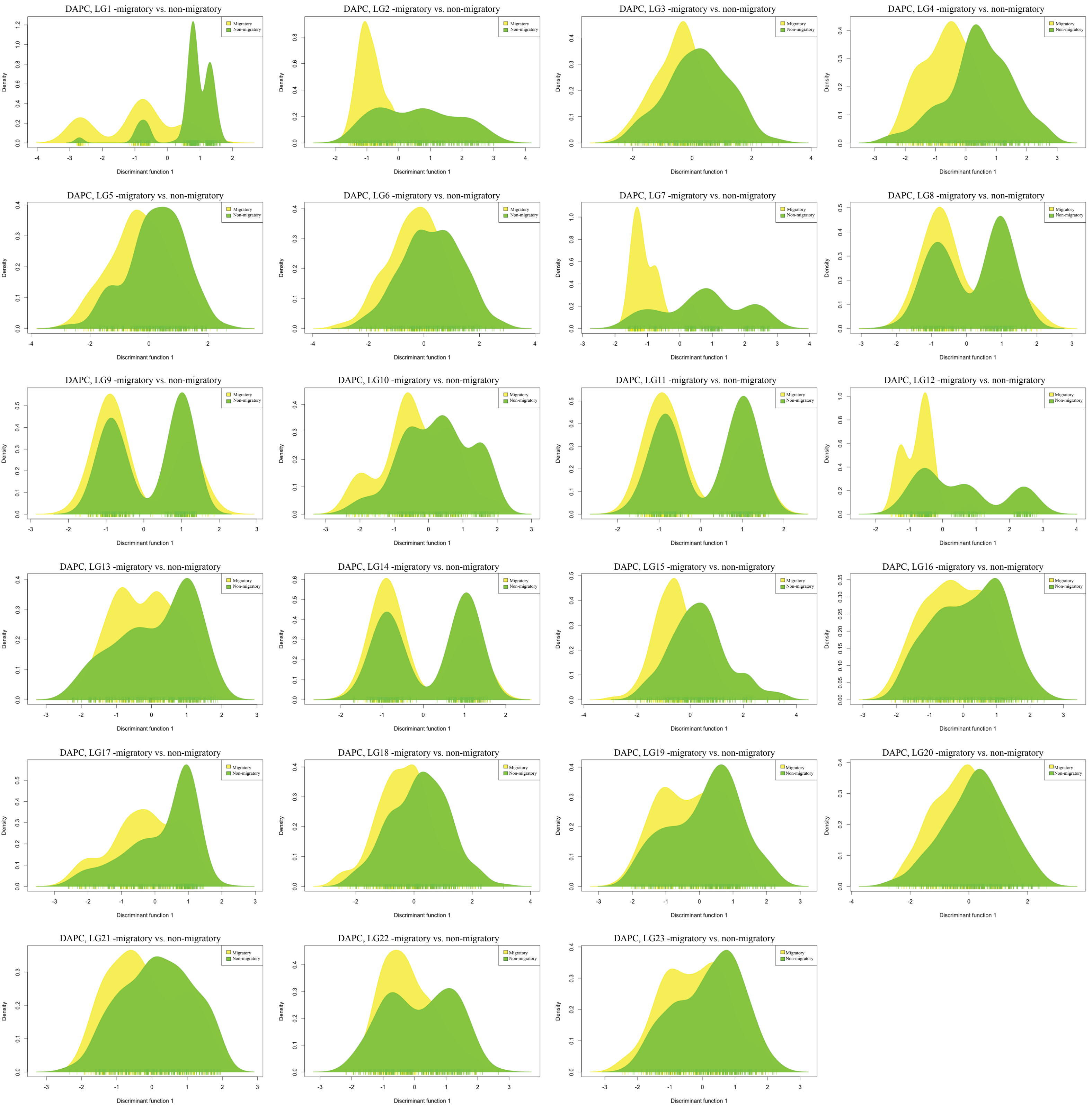

Supplementary Fig. S5. **Discriminant analysis principal component (DAPC) analysis showing the spatial relationship between migratory and non-migratory ecotypes of Atlantic cod.** (**a**) Based on all 8,165 SNPs, a distinct separation is observed between migratory and non-migratory ecotypes. (**b**) The loading plots based on the DAPC analyses shows the contribution of each SNP to the ecotype differentiation observed in plot **a**). Separate DAPC analyses show ecotype differentiation for the four divergent LGs containing inverted regions; LG1 (**c**), LG2 (**d**), LG7 (**e**) and LG12 (**f**). Results for the DAPC analyses for all LGs separate are shown in (**g**). For all plots, SNPs are ordered according to linkage group and position within the linkage groups along the X-axis as in Berg *et al.* (2016). The analyses are based on n.pca = 2 and n.da = 2, calculated in ADEGENET. The migratory group consists of Can-N_PB (Placentia Bay), Can-N_SG (Southern Gulf of St. Lawrence), Ice_F (Iceland Frontal) and NEAC (Northeast Arctic cod) while the non-migratory group consists of Can-S_SB (Sambro), Can-S_GM (Gulf of Maine), Can-S_BB (Browns Bank), Ice_C (Iceland Coastal) and NCC (Norwegian coastal cod).

Northwest Atlantic outlier pattern

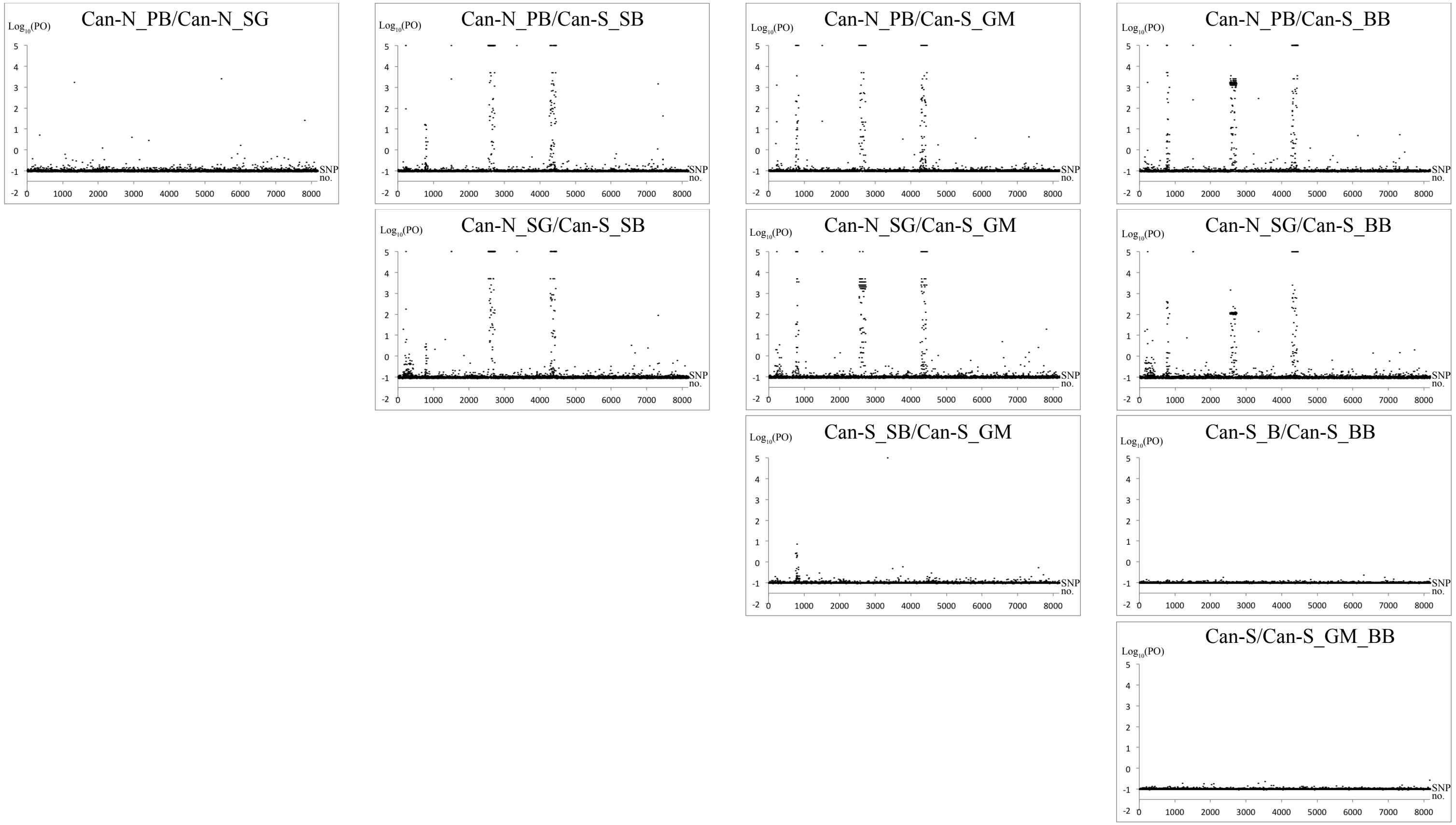

Trans-Atlantic outlier pattern

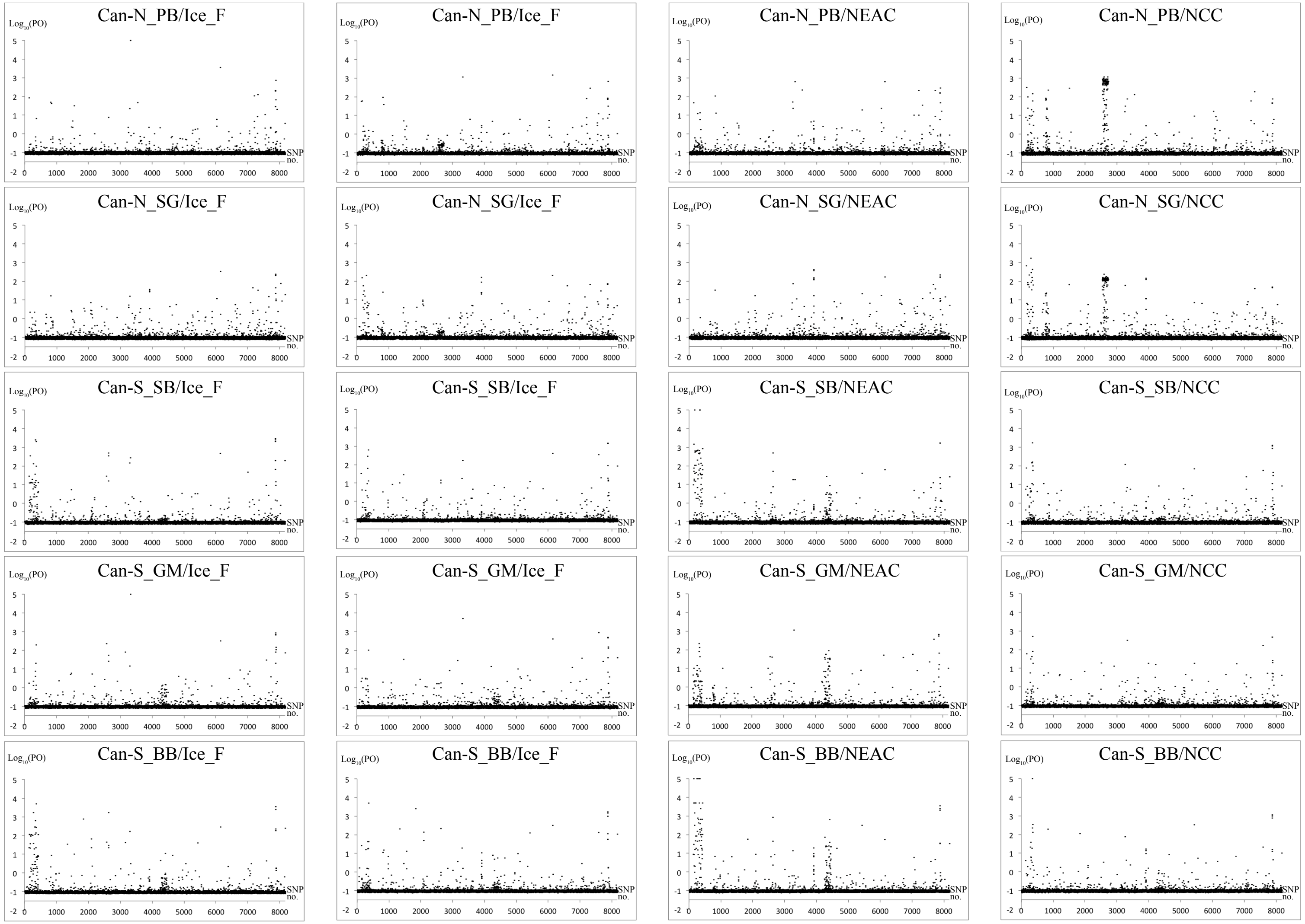

Northeast Atlantic outlier pattern

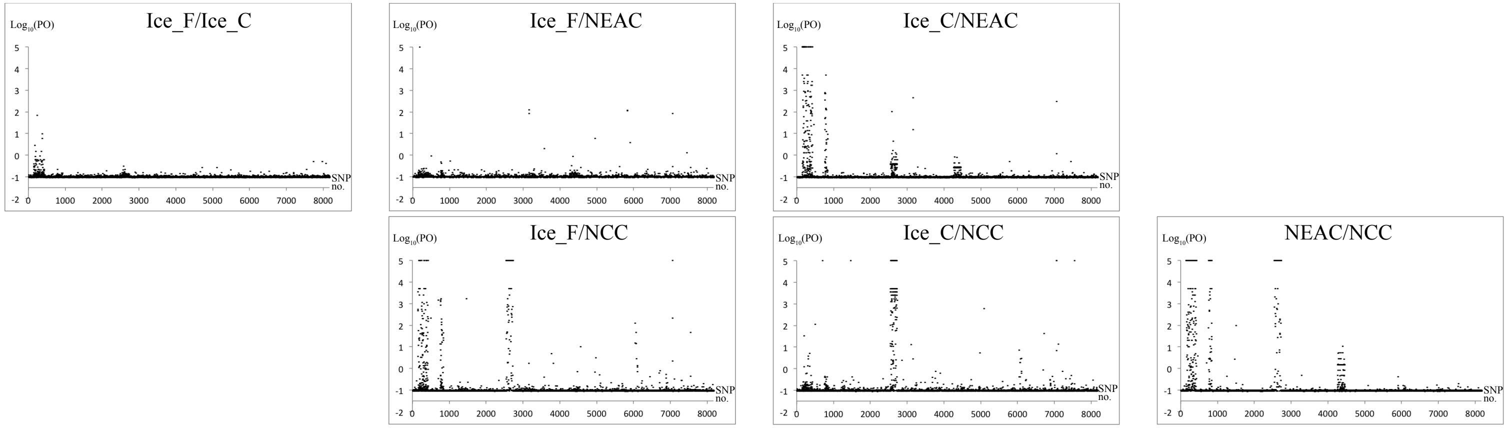

Outlier pattern by region

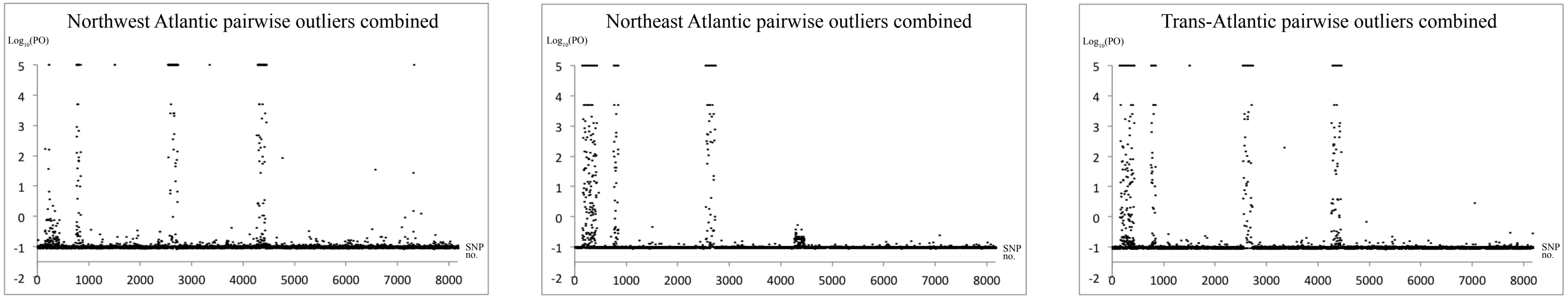

Supplementary Fig. S6. **Manhattan plots, visualizing the pairwise outlier patterns between all Atlantic cod populations in this study.** The outlier plots are based on median $\log_{10}$ (PO) values from 10 independent runs of BAYESCAN. SNPs are plotted according to linkage group and position within the linkage groups along the X-axis as in Berg *et al.* (2016). Can-N_PB = Placentia Bay, Can-N_SG = Southern Gulf of St. Lawrence, Can-S_SB = Sambro, Can-S_GM = Gulf of Maine, Can-S_BB = Browns Bank, Ice_F = Iceland Frontal, Ice_C = Iceland Coastal, NEAC = Northeast Arctic cod, NCC = Norwegian coastal cod. For visualization purpose, maximum $\log_{10}$ (PO) values are set to 5 (all underlying values are found in Supplementary Table S2).

a)

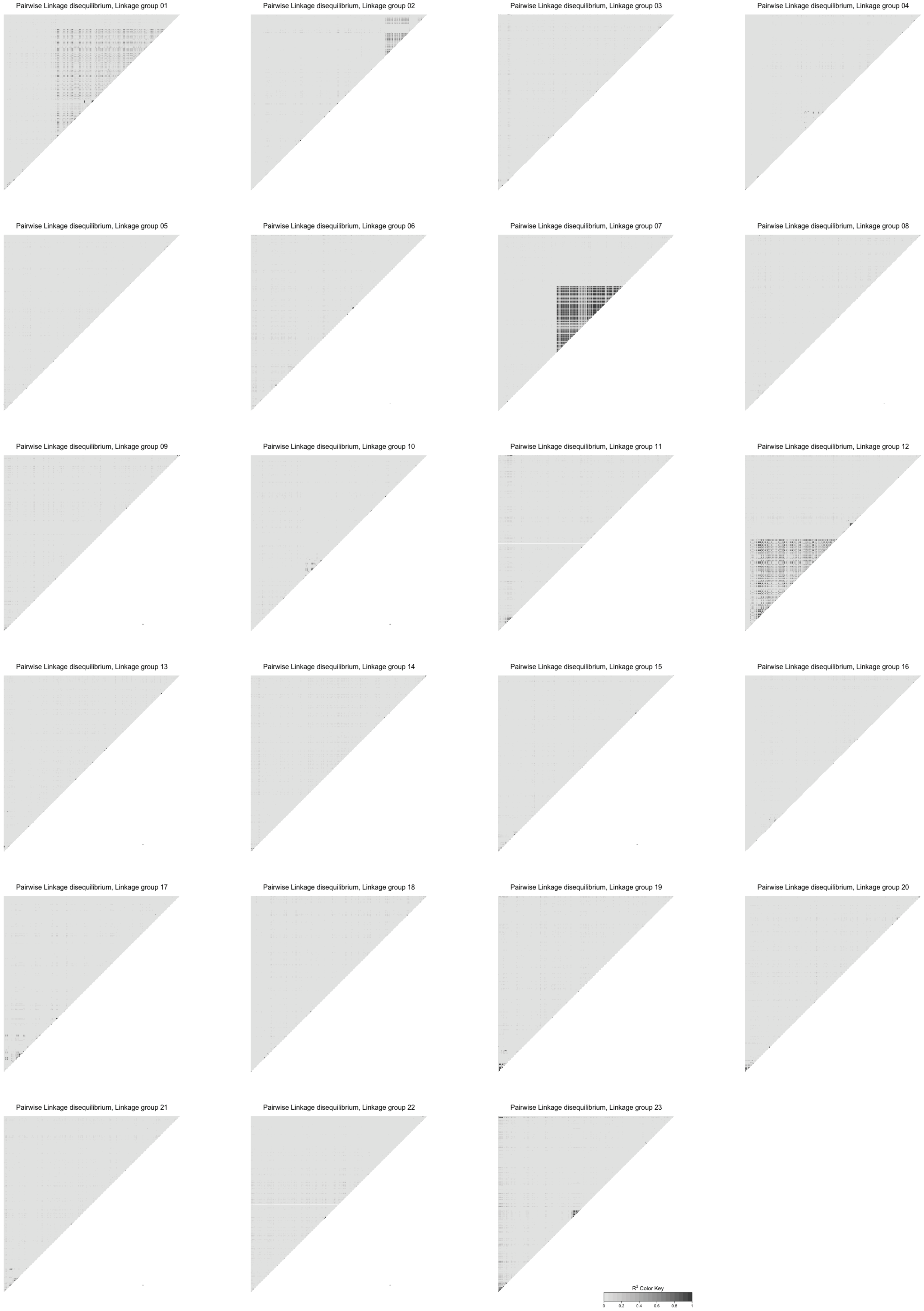

Pairwise Linkage disequilibrium, Linkage group 01
Pairwise Linkage disequilibrium, Linkage group 02
Pairwise Linkage disequilibrium, Linkage group 03
Pairwise Linkage disequilibrium, Linkage group 04
Pairwise Linkage disequilibrium, Linkage group 05
Pairwise Linkage disequilibrium, Linkage group 06
Pairwise Linkage disequilibrium, Linkage group 07
Pairwise Linkage disequilibrium, Linkage group 08
Pairwise Linkage disequilibrium, Linkage group 09
Pairwise Linkage disequilibrium, Linkage group 10
Pairwise Linkage disequilibrium, Linkage group 11
Pairwise Linkage disequilibrium, Linkage group 12
Pairwise Linkage disequilibrium, Linkage group 13
Pairwise Linkage disequilibrium, Linkage group 14
Pairwise Linkage disequilibrium, Linkage group 15
Pairwise Linkage disequilibrium, Linkage group 16
Pairwise Linkage disequilibrium, Linkage group 17
Pairwise Linkage disequilibrium, Linkage group 18
Pairwise Linkage disequilibrium, Linkage group 19
Pairwise Linkage disequilibrium, Linkage group 20
Pairwise Linkage disequilibrium, Linkage group 21
Pairwise Linkage disequilibrium, Linkage group 22
Pairwise Linkage disequilibrium, Linkage group 23
R^2 Color Key
0 0.2 0.4 0.6 0.8 1

b)

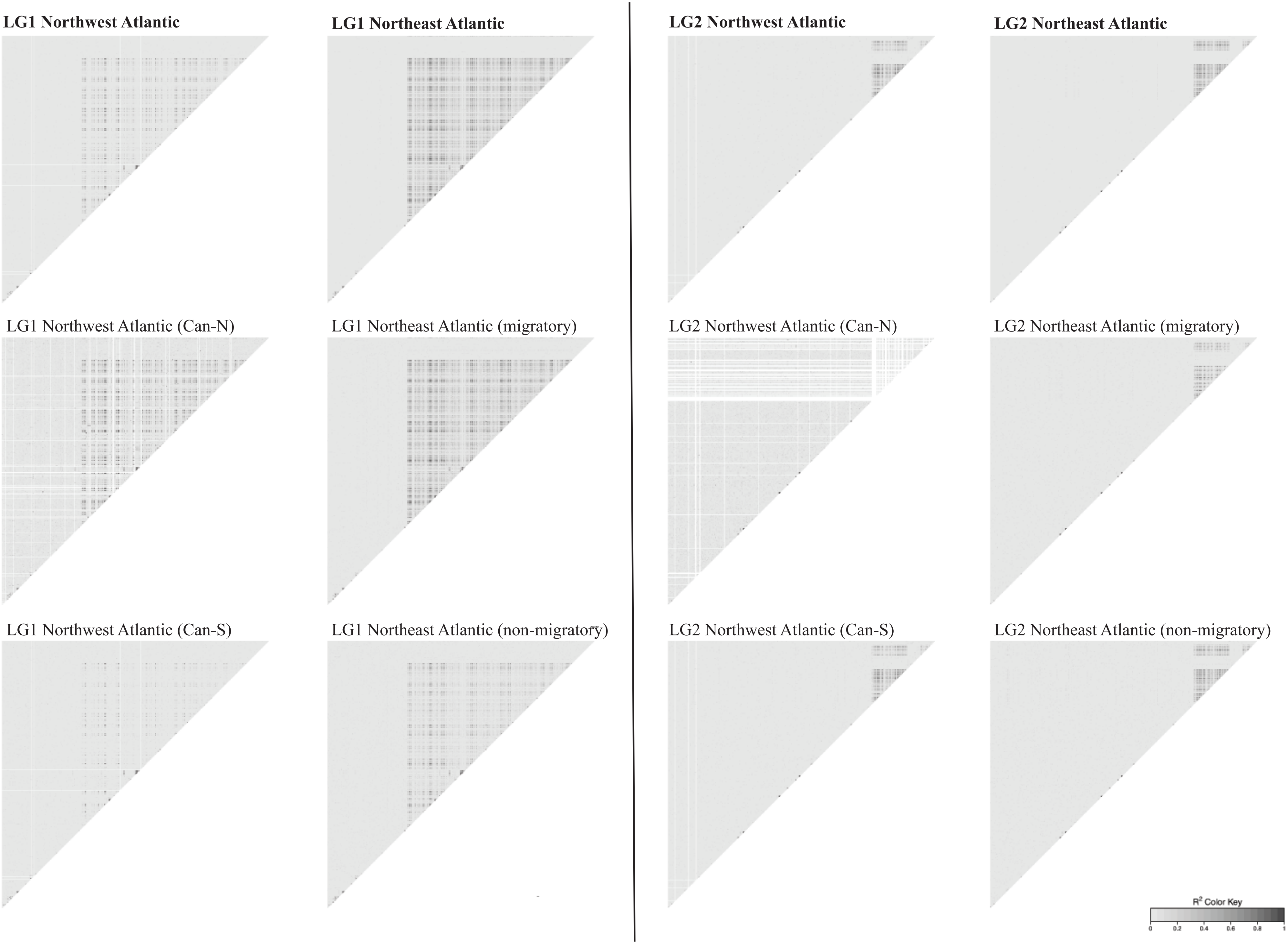
LG1 Northwest Atlantic
LG1 Northeast Atlantic
LG2 Northwest Atlantic
LG2 Northeast Atlantic
LG1 Northwest Atlantic (Can-N)
LG1 Northeast Atlantic (migratory)
LG2 Northwest Atlantic (Can-N)
LG2 Northeast Atlantic (migratory)
LG1 Northwest Atlantic (Can-S)
LG1 Northeast Atlantic (non-migratory)
LG2 Northwest Atlantic (Can-S)
LG2 Northeast Atlantic (non-migratory)
R^2 Color Key
0
0.2
0.4
0.6
0.8
1

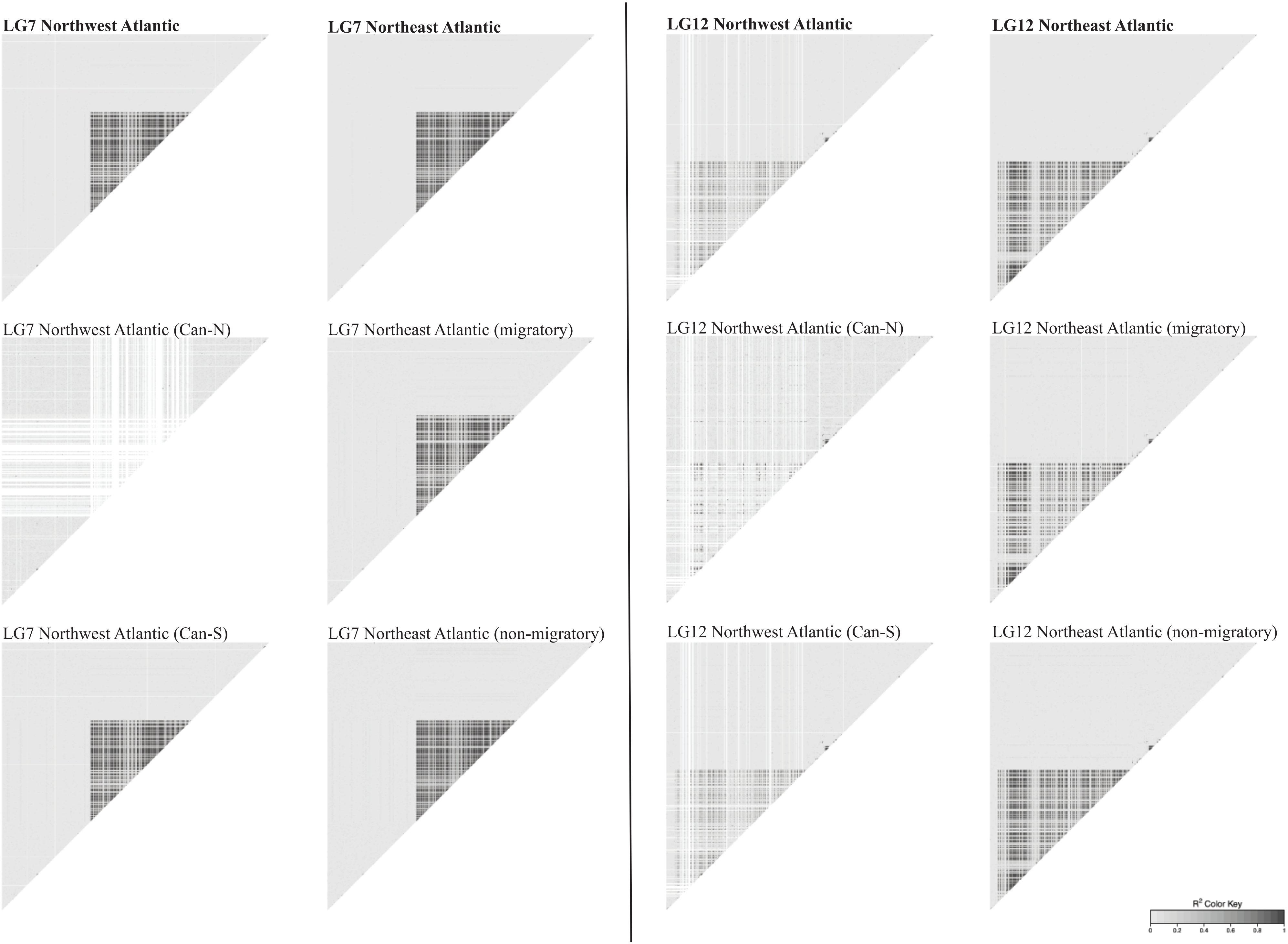
LG7 Northwest Atlantic
LG7 Northeast Atlantic
LG12 Northwest Atlantic
LG12 Northeast Atlantic
LG7 Northwest Atlantic (Can-N)
LG7 Northeast Atlantic (migratory)
LG12 Northwest Atlantic (Can-N)
LG12 Northeast Atlantic (migratory)
LG7 Northwest Atlantic (Can-S)
LG7 Northeast Atlantic (non-migratory)
LG12 Northwest Atlantic (Can-S)
LG12 Northeast Atlantic (non-migratory)
R^2 Color Key
0
0.2
0.4
0.6
0.8
1

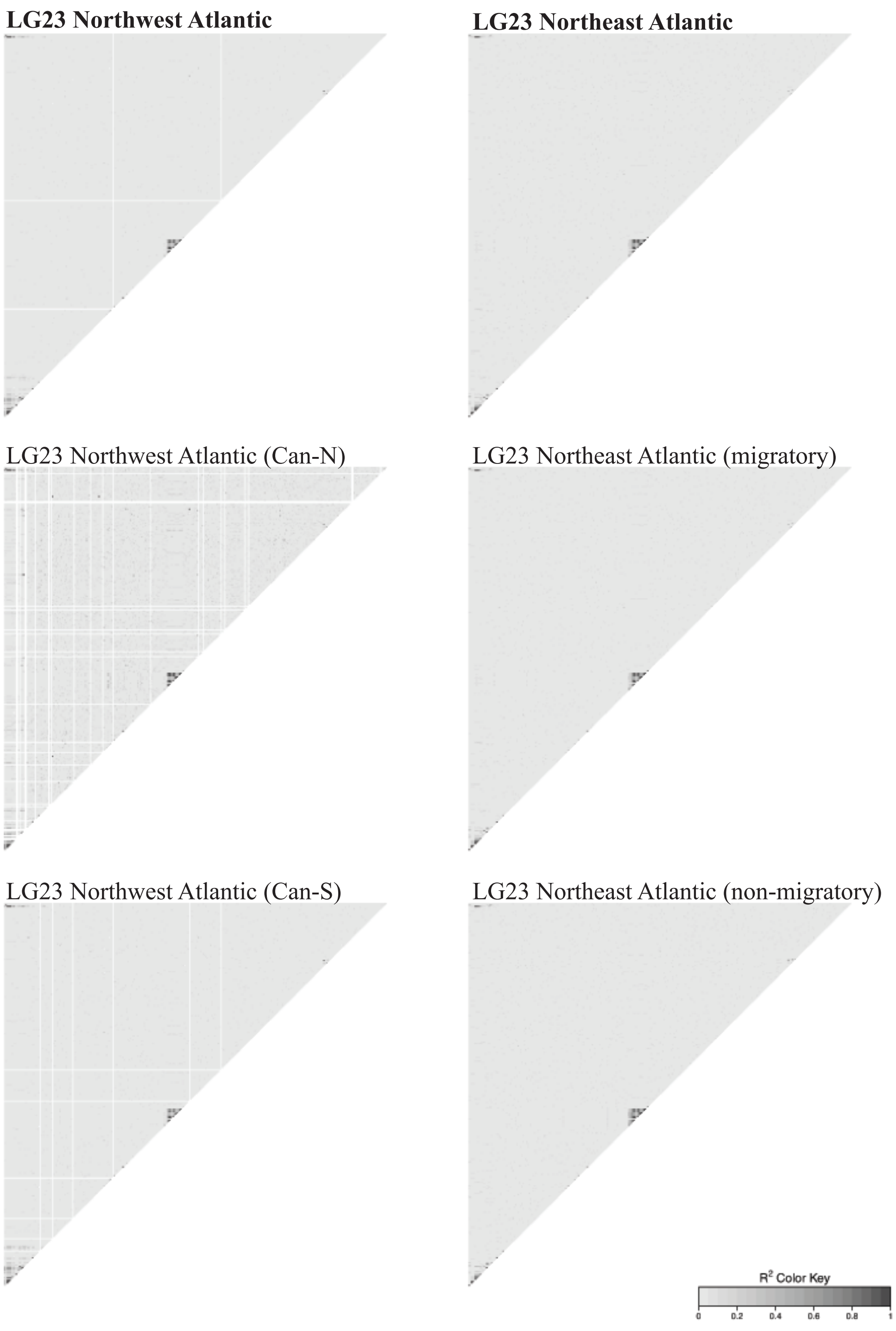

Supplementary Fig. S7. **Linkage disequilibrium (LD) in all linkage groups (LGs).** (**a**) Pair-wise LD among loci, measured as r^2, estimated in all individuals within each LG separately. (**b**) Pair-wise LD among loci, measured as r^2, estimated within each LG separately for the Can-N, migratory, Can-S and non-migratory ecotypes. SNPs are ordered according to linkage group and position within the linkage groups as in Berg *et al.* (2016). Can-N (Can-N_PB = Placentia Bay, Can-N_SG = Southern Gulf of St. Lawrence), Can-S (Can-S_SB = Sambro, Can-S_GM = Gulf of Maine, Can-S_BB = Browns Bank), migratory (Ice_F = Iceland Frontal, NEAC = Northeast Arctic cod), non-migratory (Ice_C = Iceland Coastal, NCC = Norwegian coastal cod).
